# Comprehensive Transcriptomic Investigation of Rett Syndrome Reveals Increasing Complexity Trends from Induced Pluripotent Stem Cells to Neurons with Implications for Enriched Pathways

**DOI:** 10.1021/acsomega.3c06448

**Published:** 2023-11-08

**Authors:** Yusuf Caglar Odabasi, Sena Yanasik, Pelin Saglam-Metiner, Yasin Kaymaz, Ozlem Yesil-Celiktas

**Affiliations:** Department of Bioengineering, Faculty of Engineering, Ege University, Izmir 35100, Turkey

## Abstract

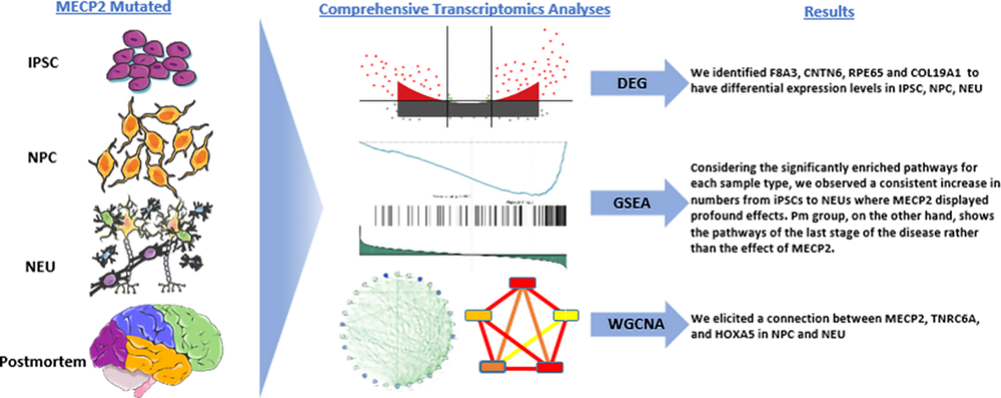

Rett syndrome (RTT) is a rare genetic neurodevelopmental disorder that has no cure apart from symptomatic treatments. While intense research efforts are required to fulfill this unmet need, the fundamental challenge is to obtain sufficient patient data. In this study, we used human transcriptomic data of four different sample types from RTT patients including induced pluripotent stem cells, differentiated neural progenitor cells, differentiated neurons, and postmortem brain tissues with an increasing in vivo-like complexity to unveil specific trends in gene expressions across the samples. Based on DEG analysis, we identified F8A3, CNTN6, RPE65, and COL19A1 to have differential expression levels in three sample types and also observed previously reported genes such as MECP2, FOXG1, CACNA1G, SATB2, GABBR2, MEF2C, KCNJ10, and CUX2 in our study. Considering the significantly enriched pathways for each sample type, we observed a consistent increase in numbers from iPSCs to NEUs where MECP2 displayed profound effects. We also validated our GSEA results by using single-cell RNA-seq data. In WGCNA, we elicited a connection among MECP2, TNRC6A, and HOXA5. Our findings highlight the utility of transcriptomic analyses to determine genes that might lead to therapeutic strategies.

## Introduction

The molecular mechanisms of neurodevelopmental diseases (NDDs) are dependent on many biological systems, and they have a complex structure since they are mostly caused by central nervous system (CNS) disorders. Thus, it is difficult to analyze the pathology of NDDs and to investigate possible treatment methods.^1^ The development of preclinical models for NDDs for which there is no proven treatment yet and the investigation of their genetic nature contributes to potential preclinical drug trials. In addition to preclinical models, combining different system analysis methods such as systems biology is also required to identify effective therapeutic targets. Systems biology is often used to examine the gene pathway and gene disease associations derived from genomic data. Investigating such interactions in relation to brain structure and function provides great convenience in deciphering the mechanism of NDDs.^2^ Within this scope, the explanation of the genetic nature of NDDs and the study of behavioral outcomes associated with brain development are recapitulated by preclinical models that summarize normal maturation trajectories.^3^

Rett syndrome (RTT), which was defined by Andreas Rett in 1966, is a rare NDD that mostly occurs in female patients, and is generally observed as a loss of hand-arm coordination, speech difficulties, irregular growth in the head structure, intestinal problems and respiratory irregularities.^4−6^ More than 95% of classic RTT cases are caused by a mutation in the MECP2 gene, which is mostly expressed in the brain and responsible for the expression of thousands of genes.^7,8^ In many genetic studies with RTT patients, genes associated with the pathology have been the main focus, and genes such as FOXG1, CDKL5, and MEF2C have been reported to be linked with addition of RTT to MECP2. However, no consensus has been reached on the mentioned genes for regulatory contribution like MECP2.^9^ Although CDKL5 mutation associated with early seizures, mental regression and wide forehead formation, is linked with RTT at first, then was separated from RTT due to early seizures (at 3 months of age).^10^ FOXG1 mutation, which is less common than CDKL5 in RTT patients, causes changes in early brain development and is considered a separate syndrome.^11^ In the case of MEF2C mutation in which mental retardation, seizures and cerebral deformation are observed, MECP2 expression is also decreased, thus it is associated with RTT.^12^ Therefore, the genetic mechanism of RTT has not been fully resolved as the risk factors have still not been determined. To date, many in vivo and in vitro models have been used in which preclinical studies have been conducted for discovery, modeling, diagnosis, and treatment of RTT.^13,14^ Mouse models with mutations in the MECP2 gene are frequently used in RTT studies. However, the need to use MECP2 heterozygous female mice and the inconsistency of symptoms in mice compared to humans due to differences in gene expression limit the usability of these models.^15^ For this reason, humanized in vitro models using neural/glial cells or brain organoids originating from patient-derived induced pluripotent stem cells (iPSCs), have gained importance for obtaining transcriptomic and proteomic data for NDDs with state-of-the-art technology in the recent past.^16,17^ As human transcriptome data are difficult to find for RTT, as a rare disease, within this scope, the number of studies in which more than one cell group or brain tissue is analyzed and compared with the integration of transcriptomic data is gaining importance.^18−20^ The scope, content, and intensity of data should be sufficient and specific to human tissues in order to have better insights into the genetic level of the disease in humans. With the latest developments in next-generation sequencing (NGS) and bioinformatic tools, a large amount of data has been obtained, which has accelerated the understanding of disease mechanisms and elicited new treatment targets by analyzing the data. Many bioinformatic analysis methods and tools have been developed to make transcriptomic analysis more extensive, which are usually performed by comparing control and disease groups at gene expression levels. Differentially expressed genes (DEG) analysis is the most basic method used to compare these two conditions.^21^ Gene set enrichment analysis (GSEA) is performed to identify the biological processes affected using changes in the expression levels of genes.^22^ Instead of focusing on individual changes in genes as in DEG analysis and GSEA, coexpression gene analysis is performed by weighted gene coexpression network analysis (WGCNA) to detect genes that exhibit group activity.^23,24^

In this study, we used human transcriptomic data consisting of four different sample types from RTT patients including induced pluripotent stem cells (iPSCs), iPSCs differentiated neural progenitor cells (NPCs), iPSCs differentiated neurons (NEUs), and brain tissues (PMs) with an increasing in vivo-like complexity to unveil specific trends in gene expression across the samples. We performed DEG analysis, GSEA, and WGCNA on all transcriptomic data to identify novel target genes at the genetic level.

## Results

**Figure 1:**
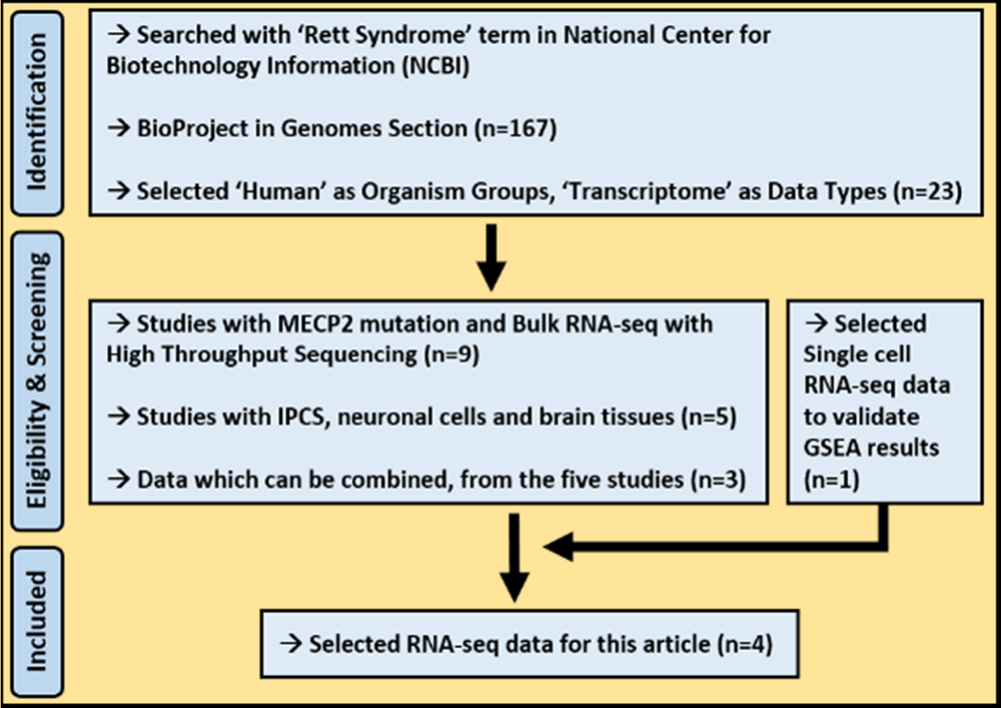
Diagram for the study cohort selection procedure.

**Figure 2:**
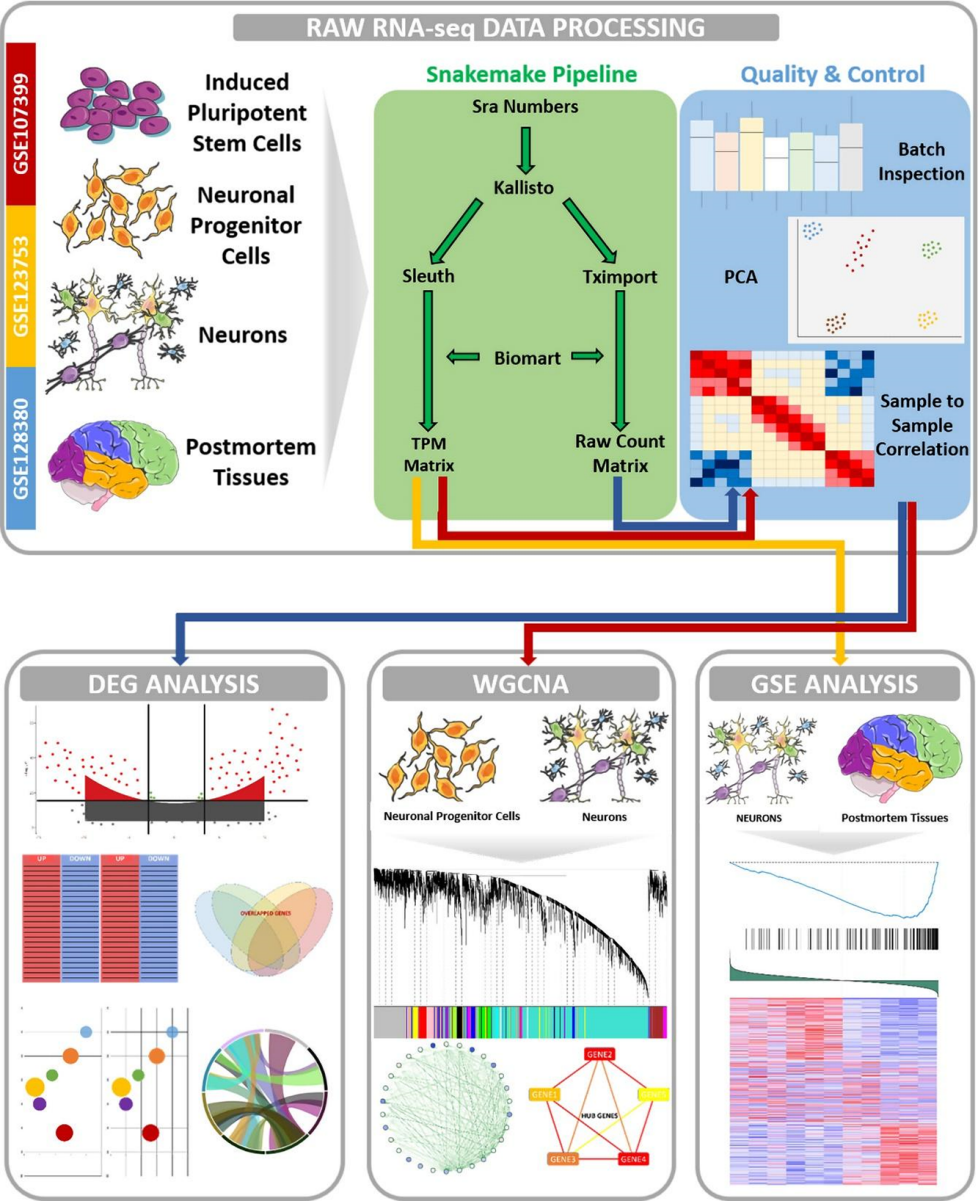
The overall study design and representative analysis scheme. Upper panel: obtaining raw RNA-seq data of iPSC, NPC, NEU, and PM tissues from three publicly available data sets (left section), gene expression quantifications with Kallisto raw sequencing read pseudoaligner software implemented as a snakemake pipeline (middle section), and quality control steps including batch effect inspection, PCA, and sample to sample correlation analysis (right section). Lower panel: three simultaneous analyses. The first is to determine differentially expressed genes and ontology analysis (left section), the second is to construct gene coexpression networks using the WGCNA approach with NPC and NEU samples (middle section), and the third is to conduct a gene set enrichment analysis with NEU and PM samples (right section) (Figure 2 was partly generated using Servier Medical Art, provided by Servier, licensed under a Creative Commons Attribution 3.0 unported license).

To investigate RTT with respect to transcriptomic changes as opposed to normal tissue counterparts, we sought to re-examine bulk RNA sequencing data sets from the National Center for Biotechnology Information (NCBI). Within the scope of data set research, 23 studies on “Rett Syndrome” were found in NCBI, and three different Bulk RNA-seq data (GSE128380, GSE107399, and GSE123753) with MECP2 mutations were identified that could be analyzed together. In addition, single cell RNA-seq data from fused cortex + ganglionic eminence organoids (GSE165577) with MECP2 mutation was used for GSEA validation (Figure 1). We meticulously inspected each data set to determine whether they can be compared and contrasted in the context of our study. We initially aimed to process each data set to bring them to a comparable form by accounting for different batches. The overall study design can be seen in Figure 2. Briefly, transcriptomic data of iPSCs, NPCs, NEUs, and PM tissues were collected from NCBI GEO database and prepared for analysis to examine the developmental mechanism of RTT. The collected raw sequencing data were processed using a custom analysis pipeline (detailed in methods) to obtain uniformity. Following the preprocessing and quality controls, we examined each sample for certain inclusion criteria using sample-to-sample correlations and PCA.

We excluded samples with low sequencing depth compared with their counterparts and preserved the ones with similar gene expression distributions. We conducted three separate analyses, namely differential gene expression analysis (DGE), de novo coexpression network analysis (WGCNA), and enrichment of predefined gene sets based on transcriptomic differences (GSEA). Our main purpose was to determine transcriptomic differences between samples from patients diagnosed with RTT and samples from healthy individuals with respect to various cell-tissue types with an increasing in vivo-like complexity. As a result, we intended to associate differential usage of individual genes or gene sets (coexpression modules, signaling cascades, or pathways) across conditions with tissue states. We obtained a total of 82 short read RNA sequencing raw data. The sample collection consisted of bulk RNA sequencing data sets of (i) post-mortem brain tissue samples from four females clinically diagnosed with RTT and four age-matched female healthy donors (GSE128380), (ii) iPSCs from two clinically diagnosed with RTT patients, NPC and NEU samples derived from mutant/isogenic iPSCs and two post-mortem brain tissue samples with their age-matched RTT/healthy donors (GSE107399), and also (iii) NPC and NEU samples derived from mutant/isogenic iPSCs (GSE123753) (Table 1). The detailed description of the data is shown in Table S1.

**Table 1:** List of Studies and Their Respective Sample Cohorts Included in Our Analysis^a^

**study GEO ID**	**disease status**	**sample type**	**MECP2 mutations^a^**
**GSE107399**^b^	CTRL (*N* = 16)	post-mortem, iPSC, NPC, Neù	
	RTT (*N* = 14)	post-mortem, iPSC, NPC, Neu	705delG, 1461A > G, splice site variant (c.378A > G)
**GSE123753**^c^	CTRL (*N* = 24)	iPSC, NPC, Neu	
	RTT (*N* = 12)	NPC, Neu	exons 3–4 deletion
**GSE128380^d^**	CTRL (*N* = 8)	post-mortem	
	RTT (*N* = 8)	post-mortem	c.473C > T, exon deletion

a These variants are not uniform across samples. Healthy samples (isogenic controls for cells, healthy donors for postmortem tissues); CTRL, Rett syndrome samples; RTT.

b Ref. (25).

c Ref (26).

d Ref (27).

We aligned short sequencing reads to human reference transcriptome using a pseudo aligner, Kallisto, and filtered out noncoding transcripts to solely focus on protein coding genes which reduced the number of genes to 19,315. We used the union gene model when counting reads per gene and merged all possible transcript isoforms.

In order to reduce the noise in the data matrix and obtain unbiased expression differences between Ctrl and RTT, genes with average expression values greater than 1.0 TPM were selected. We examined possible batch effects across study cohorts and sample sets using expression distributions and principal component analysis (PCA) (Figure S1A). In particular, the PCA demonstrated samples separate from each other based on experiment batches rather than tissue or condition, which indicated a need for the batch effect correction (Figure S1B). Therefore, we proceeded with batch correction on the expression data using ComBat() function from an R package called “SVA” following the log2 transformation of normalized data. After the batch correction, we re-examined the variance structure of the expression data across samples to ensure comparability in terms of desired conditions. In addition to the PCA, we created a sample-to-sample Pearson correlation heatmap of our data to inspect similarities of each sample to others. As a result, we decided to exclude NPC samples from one of the studies (GSE107399) due to inconsistent expression distribution across the samples (Figure S1C). While we used raw count data of genes when conducting differential expression tests of individual genes by accounting for different batches as latent factors, we performed WGCNA and GSEA on TPM normalized gene expression data.

### Four Genes Have Similar Expression Differences across Sample Types and Common GO Terms with MECP2

**Figure 3:**
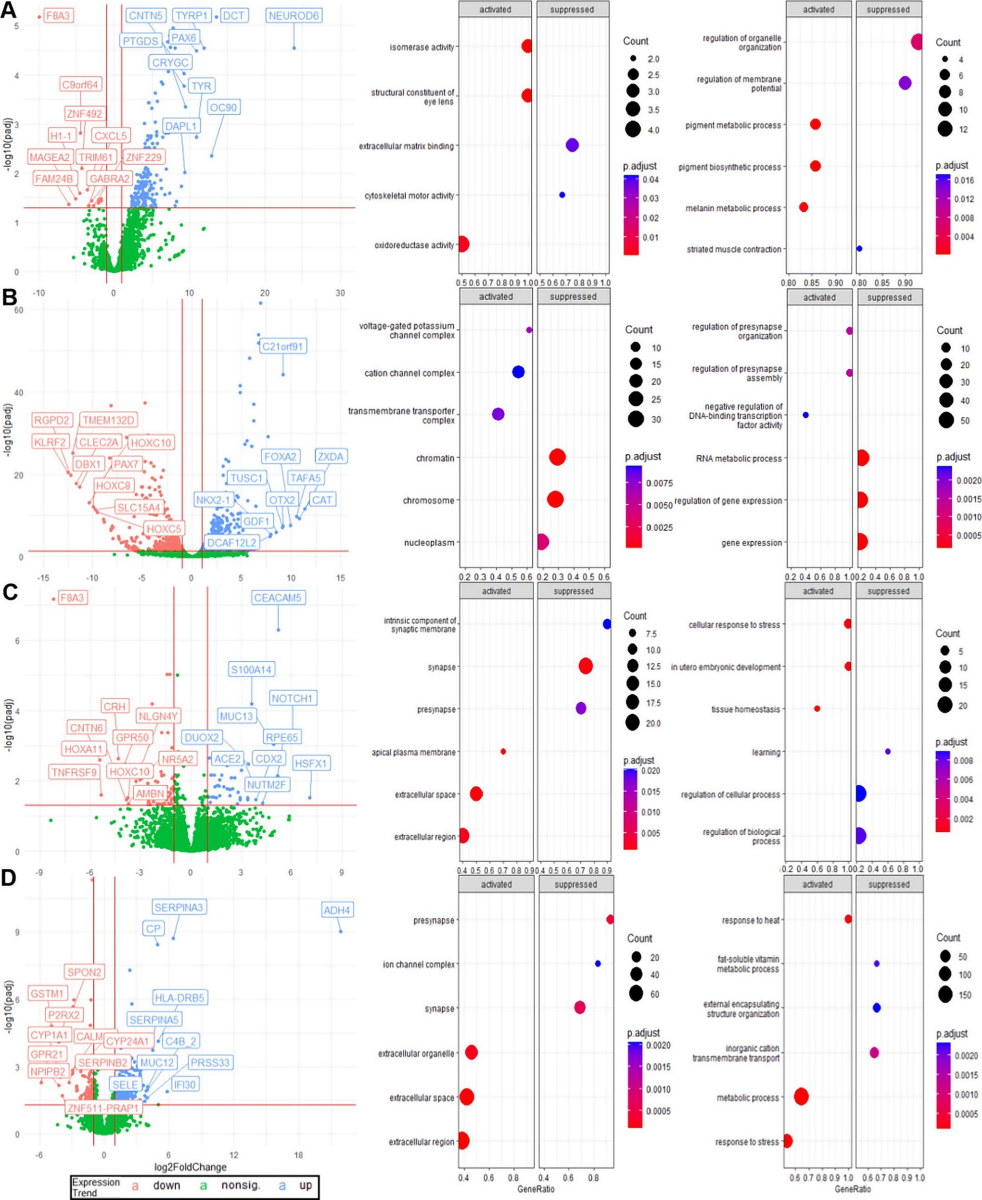
Differentially expressed genes between CTRL and RTT for the four sample types and their top 3 GO terms. The locations of four prominent genes in top 3 GO terms were examined. (A) 16 downregulated 197 upregulated genes (*p* adj. value ≤ 0.05 and log2 fc > 1), including F8A3 and RPE65, were detected in iPSC. The GO category MF (left dotplot) and BP (right dotplot) were shown for iPSC as they contain RPE65. (B) There are 443 downregulated and 461 upregulated genes identified in NPC, including RPE65, F8A3, CNTN6, and COL19A1. Since MECP2 and F8A3 have common GO terms and are observed in only two GO categories, CC (left dotplot) and BP (right dotplot) were shown. (C) For NEU^53^ downregulated and 96 upregulated genes, including also four prominent genes like at NPC were detected. Only the CC (left dotplot) and BP (right dotplot) were shown because MECP2, F8A3, CNTN6, and RPE65 were observed in these GO categories. (D) There are 288 downregulated and 197 upregulated genes in PM, including CNTN6 and COL19A1. CC (left dotplot) and BP (right dotplot) GO categories were shown.

We initially sought to determine individual gene expression differences between samples originated from patients RTT and CTRL since we hypothesized that the fundamental transcriptomic alterations due to disease condition should be preserved through the differentiation of iPSCs toward neuronal types and eventually primary brain tissues. Therefore, we first determined differentially expressed genes between RTT samples and their normal counterparts within each sample type. We, then, compared the list of significantly differentially expressed genes to determine common genes across types. Our sample cohort formed four main types: iPSCs, NPCs, NEUs, and PMs. As a result, there were 197 up and 16 downregulated significant genes in iPSC group, 443 up and 461 downregulated genes in NPCs,^53^ up and 96 downregulated genes in NEUs and finally 288 up and 197 downregulated genes in PMs between their RTT and CTRL samples (Figure 3 and Table S2). There were no common genes that were differentially expressed in all four sample types, but we got four genes (RPE65, F8A3, CNTN6, COL19A1) differentially expressed across three different sample types.

RPE65 gene was consistently upregulated in RTT of iPSCs, NPCs, and NEUs whereas F8A3 was consistently downregulated in the same sample types. In addition, CNTN6 and COL19A1 were downregulated in RTT of all three NPCs, NEUs, and PMs (Table S3). To further investigate all differentially expressed genes, we conducted a Gene Ontology analysis and examined the relationship between four prominent genes above and MECP2 from the point of GO terms across four sample types and hub genes analysis. The first three most significant GO terms of activated (upregulated) and suppressed (downregulated) genes from each sample type are summarized in Figure 3. It supports our expectations that the disease effect increases from iPSC to NEU level since while the top three GO terms of iPSCs are not much related to RTT, we encountered nucleosome, chromosome, gene expressions and presynapse organization and assembly in GO terms that change in NPCs so we accepted NPCs as the level where RTT effects are gradually observable. Moreover, we say that NEU is the level where RTT effects are observed more since we found GO terms such as snaps, learning, and cellular regulation, where MECP2 effects are observed more. In addition to the GO analysis, when IPSC hub genes were examined, we observed that they were all upregulated and related to signaling pathways related to the tissue development (TGFB2, LTBP3), the organization of the extracellular environment (FBN1, ELN, MITF, DCN, TIMP3, TGFB2) and collagen synthesis (FBN1, COLGALT2, COL8A1, COL11A1, COL5A1, COL2A1, COL1A2, COL1A1, DCN). NPC hub genes are associated with various pathways related to neuronal signaling, development, and differentiation. They play important roles in glutamatergic synapses (GRIK2, GRIK5, GRIA3, and GRIN2D), neurotransmitter interactions (SHISA6, SHH, FOXG1, CACNG4, CACNG5, CACNG8, and PAX6), and the development of dopaminergic neurons and oligodendrocytes in the central nervous system (OTX2, ASCL1). As for NEU hub genes, the association with pathways related to transcriptional regulation by MECP2 (GATA6, MECP2, NOTCH1, LHX5, SIM1) and neuronal signaling (CRH, DDC, LGR5) including GABA signaling (GAD2, GABRA3, GRIA1, MECP2) and the transcription of neuronal ligands (CALB1, MECP2) were notable. Finally, when we examine the PM hub genes (IL15, ICAM1, SELE, TLR3, CCL2, CXCL5, CXCL10, CXCL1, HGF, IL1R1, IL1RN, SELL, CSF1, CD44, CD163), we realized that these genes are all upregulated and closely associated with pathways related to inflammation, immune responses, and cytokine signaling (Data sets S1). When we examined four overlapped genes, we observed RPE65 in the isomerase activity and the oxidoreductase activity as molecular functions (MF) and the pigment metabolic process and pigment biosynthetic process as biological processes (BP) in iPSCs, the tissue homeostasis as BP in the NEU type. While we found that F8A3 and MECP2 were associated within the nucleoplasm and MECP2 was in the other 6 GO terms for NPCs, we detected F8A3 and MECP2 in the regulation of cellular process and regulation of biological process as BP in NEUs. We also observed CNTN6 in the same BPs as MECP2 and F8A3, while we observed it in the synapse as CC with MECP2 in NEUs. The fact that we detected F8A3 and CNTN6 in common GO terms with MECP2 as a result of the analysis raises the idea that these genes can be driver genes associated with MECP2 and RTT. Lastly, we found COL19A1 in the external encapsulating structure organization as BP and CNTN6 in the presynapse and the synapse for PMs (Figure S2), so RPE65 and COL19A1 seem to be independent of MECP2 in their expression and function.

### MECP2 Appears in the Most Significant Network Modules of NPC and NEU Groups

WGCNA is used for grouping genes that have interdependent expression patterns (coregulations) in a cluster (module) and relating all identified modules with phenotype of interest. Therefore, we performed WGCNA to examine coregulated genes and their possible associations with RTT. We were able to isolate statistically significant coexpressed gene modules when comparing RTT and CTRL within NPC and NEU types while there were no significant modules detected for iPSC and PM types (Table S4). Significant modules refer to modules that are differentially expressed in RTT and we selected the modules based on the adjusted p-values (*p* < 0.05) obtained. In order to examine previously known intergene connections, gene functions, and disease relations, we performed a cross analysis using the STRING database (STRINGdb) and retrieved the connection (edges) information, using the Human Protein Atlas (HPA) to get functions and disease relations information (Data sets S2). As a result of our analysis, 8 out of 28 modules formed for NPC comparisons were significant modules whereas 13 out of 22 were significant for NEUs. When we examined the pathways associated with the significant modules in STRINGdb, module 6, which is related to neuronal system and axon development, and module 4, which is related to receptors, gene expression, and transcription, emerged for NPCs (Data sets S3). In addition, module 6 was determined as the most significant module among NPCs modules. MECP2 and F8A3 genes appeared to be significantly coregulated within module 6, while BDNF and RPE65 were connected in the module 4 network. For NEUs, module 3, which is related to gene expression and transcription, and module 6, which is related to gaba receptors and neuronal signal transmission, are recalled (Data sets S3). We found that Module 3, besides being the most significant module, includes MECP2 and CNTN6. Therefore, we constructed and analyzed the network model of module 6 of NPCs and module 3 of NEUs in order to better identify possible driver genes that may be effective in RTT (Data sets S2).

### MECP2 Links with More Transcriptomic Factors in the NEU and HOX Group, and TNRC6A and UNC5D Show Similar Trends in Both NPC and NEU

**Figure 4:**
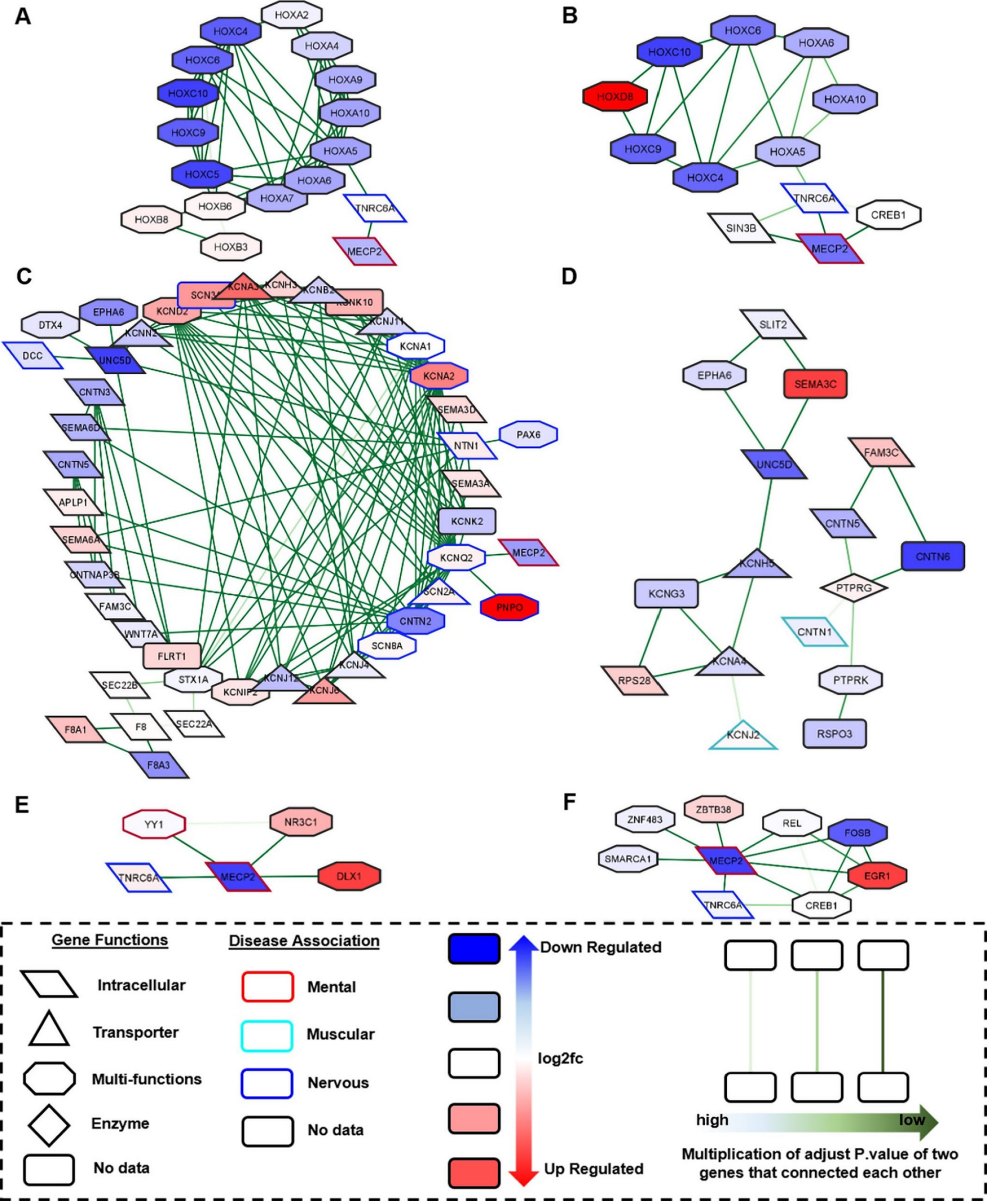
Construction of the correlation network. Relevant genes were extracted from the most significant modules’ networks. Left side of the figure shows the subnetwork of NPC module 6, and the right side is for NEU module 3′s subnetworks. The bottom panel shows the features of the networks. The shapes of nodes indicate gene functions, the frame colors of nodes indicate disease association, the nodes colors change according to log2 fc values and the edge color change based on multiplication of adjusted *P*values of two genes that connect to each other. (A, B) Relationships between HOX genes groups and MECP2 in NPC and NEU. (C, D) Differences of KCN/SCN genes groups between NPC and NEU. (E, F) Transcription factors connected to MECP2 in NPC and NEU.

We examined our network models by considering gene function, disease association, and connection information with MECP2 to choose possible effective genes in RTT. We observed a decrease in expression levels in the **HOXA/B/C** (HOXA2/4/5/6/7/9/10, HOXB3/6/8, HOXC4/5/6/9/10) gene groups, which were found to bind to **MECP2** via **TNRC6A**. MECP2 is associated with mental diseases, while TNRC6A is associated with nervous diseases in our network model (Figure 4A). In addition, **KCN/SCN** (KCNA1/2,/3, KCNB2, KCND2, KCNH3, KCNIP2, KCNJ4/6/11/12, KCNK2/10, KCNN2, KCNQ2, and SCN2*A*/3A/8A) groups linked to the MECP2 via KCNQ2 in this network. Heterogeneity had been detected in both the expression levels and functions of the genes. In addition, KCNA1/2/3, KCND2, KCNQ2, SCN2*A*/3A/8A, CACNA1G, and EGFR genes were found to be hub genes of network (Figure S3A). We detected **F8A3**, which was one of the prominent genes in our DEG analysis, to be linked to only two genes (F8 and F8A1) and indirectly linked to the KCN/SCN gene group (Figure 4C). Moreover, it was determined that the MECP2 gene connects with 14 different genes in the network. Out of 14 genes, **DLX1**, **NR3C1**, and **YY1** are transcription factors, and YY1 is also associated with mental diseases (Figure 4E). We also examined NPC module 4 as it is associated with gene expression and transcription pathways and found that BDNF and RPE65 are involved in this module. BDNF, stimulates intracellular signaling and controls neurogenesis that is critical for neuronal survival, morphogenesis, and plasticity,^28^ and has connections with 45 genes in this module. No notable change was observed in the expression level of the BDNF. RPE65 is also an enzyme functioning intracellularly, associated with nervous diseases including retinal disorders.^29^ RPE65 has connections with nine genes and an increased expression level (Data sets S2). With these findings, while reconsolidating the hypothesis that RPE65 is independent of MECP2, we understand that the same is true for BDNF.

Since we detected the same features of the NPC’s HOX group in NEU module 3, it supports the hypothesis that the HOX group may be an important gene group for RTT (Figure 4B). Different from the KCN/SCN group in NPC module 6, we detected only four genes (**KCNA4**, **KCNG3**, **KCNHA5**, and **KCNJ2**) in module 3 of the NEUs. However, in both NPCs and NEUs modules, the KCN group is linked to **UNC5D** and **EPHA6**. In NPC, UNC5D was together with EPHA6, DCC, NTN1, FLRT1, and SLITRK3, while in the NEU, it was found to be connected with SLIT2 via EPHA6 and SEMA3C. Although the KCN/SCN group is the hub gene group in NPC module 6, only four genes belonging to the KCN group were found in NEU module 3. While there is a decrease in the expression levels of CNTN6 and CNTN5, no significant change was observed in CNTN1 (Figure 4D). We detected 18 genes connected to MECP2 and 7 of these genes (**CREB1**, **FOSB**, **EGR1**, **REL**, **ZBTB38**, **SMARCA1**, and **ZNF483**) are intracellular transcription factors in NEU module 3. While we observed an increase in the expression level of EGR1,we observed a decrease in the expression level of FOSB (Figure 4F).

Apart from these, **HIST1H4F**, **SRSF2**, **SLU7**, **PABPN1**, **CREB1**, **CLP1**, **SYMPK**, **CPSF7**, **MECP2**, and **SETD7** were identified as hub genes of this module (Figure S3B). We detected the GABR (GABRA1/2/3/4, GABRB1, GABRG3) group as highlighted genes in NEU module 6, which is related to pathways such as GABA receptors, neuronal system, and neuronal signal transmission. A decrease was observed in the expression levels of other genes except GABRA1. The hub genes of the module are the GABR group, GLRA3, BEST2, HAP1, and SNRPD3 (Figure S3C). Our detection of this module belonging to the GABR group supports the neuronal system and signal transmission disorders observed in RTT.

#### NEU Is the Level Where Mutant MECP2 Effects Are Best Observed in Enriched Pathways

**Figure 5:**
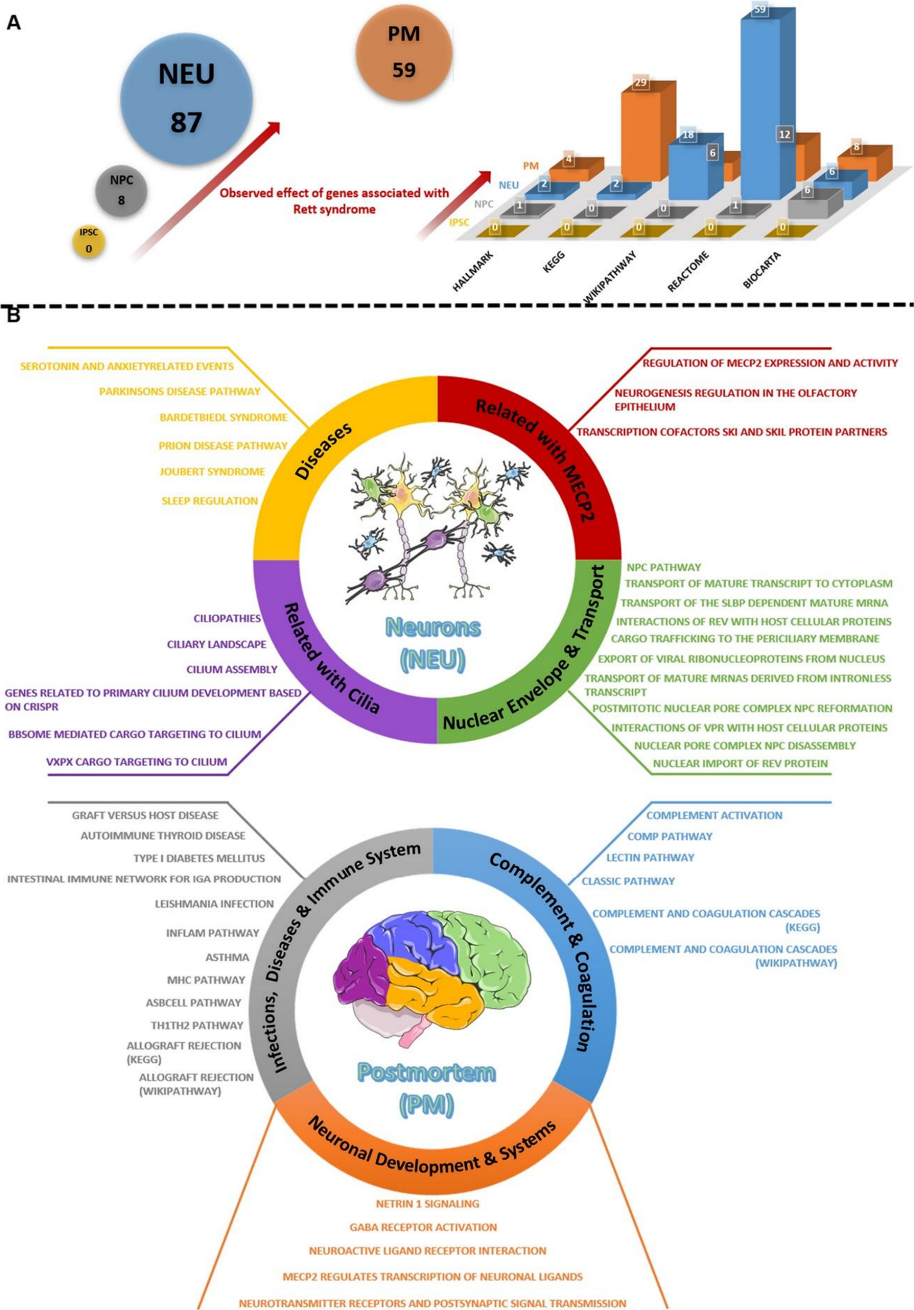
Gene set enrichment analysis of iPSC, NPC, NEU, and PM using canonical pathways from MSigDB.(A) Number of enriched pathways from iPSC to PM and their distribution between the databases. An increase is observed from iPSC to NEU, and we kept apart the PM since iPSC, NPC, and NEU are cell groups while PM is tissue. Number of detected pathways for iPSC, NPC, and NEU are 0, 8, 87, respectively, while 59 pathways are detected for PM. (B) Among the 87 pathways identified in the NEU group, we selected those related to similar biological and molecular systems and divided them into 4 groups. The same procedure was performed in PM and 3 groups were created (Figure 5 was partly generated using Servier Medical Art, provided by Servier, licensed under a Creative Commons Attribution 3.0 unported license).

In order to better understand the developmental mechanism of RTT, we identified the affected pathways for each sample type by GSEA. Pathways from Reactome, WikiPathway, KEGG, Biocarta and Hallmark databases were taken from MSigDB and used as reference gene sets for iPSCs, NPCs, NEUs and PMs. As a result of the analysis, we have seen that the NEUs reflect the RTT effect better than the other sample types. While 87 significant pathways were identified for the NEUs, 59 pathways for the PMs and 8 pathways for the NPCs were significantly enriched. There is no statistically significant pathway detected for iPSCs (FDR value <0.25) (Figure 5A and Data sets S4). In the NPCs, 6 Biocarta (βarr Mapk Pathway, β-Arrestin Src Pathway, Biopeptides Pathway, Calcineurin Pathway, Prion Pathway, Pten Pathway), 1 Hallmark (TGF Beta Signaling) and 1 Reactome (RNA Polymerase III Transcription) pathways were significant. The pathways identified in the NPCs are involved in fundamental cellular processes such as cell growth, differentiation, proliferation, survival, migration and apoptosis, constitute the majority.^30−32^

In the NEU group, pathways that may be related to RTT were examined in four main subgroups (“Related with MECP2″, “Diseases”, “Related with Cilia” and “Nuclear Envelope & Transport”). One of the subgroups of the NEUs is “Related with MECP2″, consisting of three pathways that are about the effect of MECP2 on olfactory epithelium and ski-skil proteins. The subgroup “related with Cilia” consists of seven pathways in which cilium structure and functions are defined. The “Diseases” subgroup consists of 6 disease pathways (Parkinsons Disease, Bardet-biedl Syndrome, Prion Disease, Joubert Syndrome, Sleep regulation, Serotonin and Anxiety related events). Since Joubert Syndromes and Bardet-biedl Syndromes are associated with ciliopathy like RTT and also the common denominator of these 6 diseases and RTT has been reported as the dendritic spine differentiation.^33,34^ In the “Nuclear Envelope & Transport” subgroup, 10 pathways related to the cell membrane, nucleus-pore complex and transport between nucleus and cytoplasm were identified. In the case of MECP2 mutation, it has been stated that there is a decrease in the components of the cell membrane structure, along with deterioration and also cell proliferation is altered due to this disruption.^35,36^ In our results, four genes (NUP214, NUP98, NUP205, NUP210) common to the relevant 10 pathways were also detected in the NEU module 5 module. These results show us that RTT effects from iPSCs to NEUs are much more observable and early RTT onset is detected in NPC, as we encountered in our DEG analysis. As for the PMs, pathways that may be associated with the RTT process were evaluated in three main subgroups (“Neuronal Development & Systems”, “Complement & Coagulation” and “Infection, Diseases & Immune System”) (Figure 5B). There are 12 pathways in the “Infections, Diseases & Immune System” subgroup, and almost every pathway is related to immune response processes such as inflammation. Therefore, they have several common genes such as **HLA-DRB1**, **HLA-DPB1**, **HLA-DQA1**, **DRA**, **TNF**, and **CD40**. In the “Complement & Coagulation” subgroup, there are 6 pathways and all of the pathways are related to the immune response like the previous subgroup. Particularly, **C4A**, **C4B-2**, **CFB**, **SERPINA1**, **SERPINA5**, and **C1Q complex** (C1QA, C1QB, C1QC) genes stand out in the pathways involved in the activation of proteins in the plasma against pathogens and the regulation of blood pressure. The last subgroup “Neuronal Development & Systems” consists of five pathways. This subgroup mostly specifies netrin-1 signal and GABA receptor systems.

**Figure 6:**
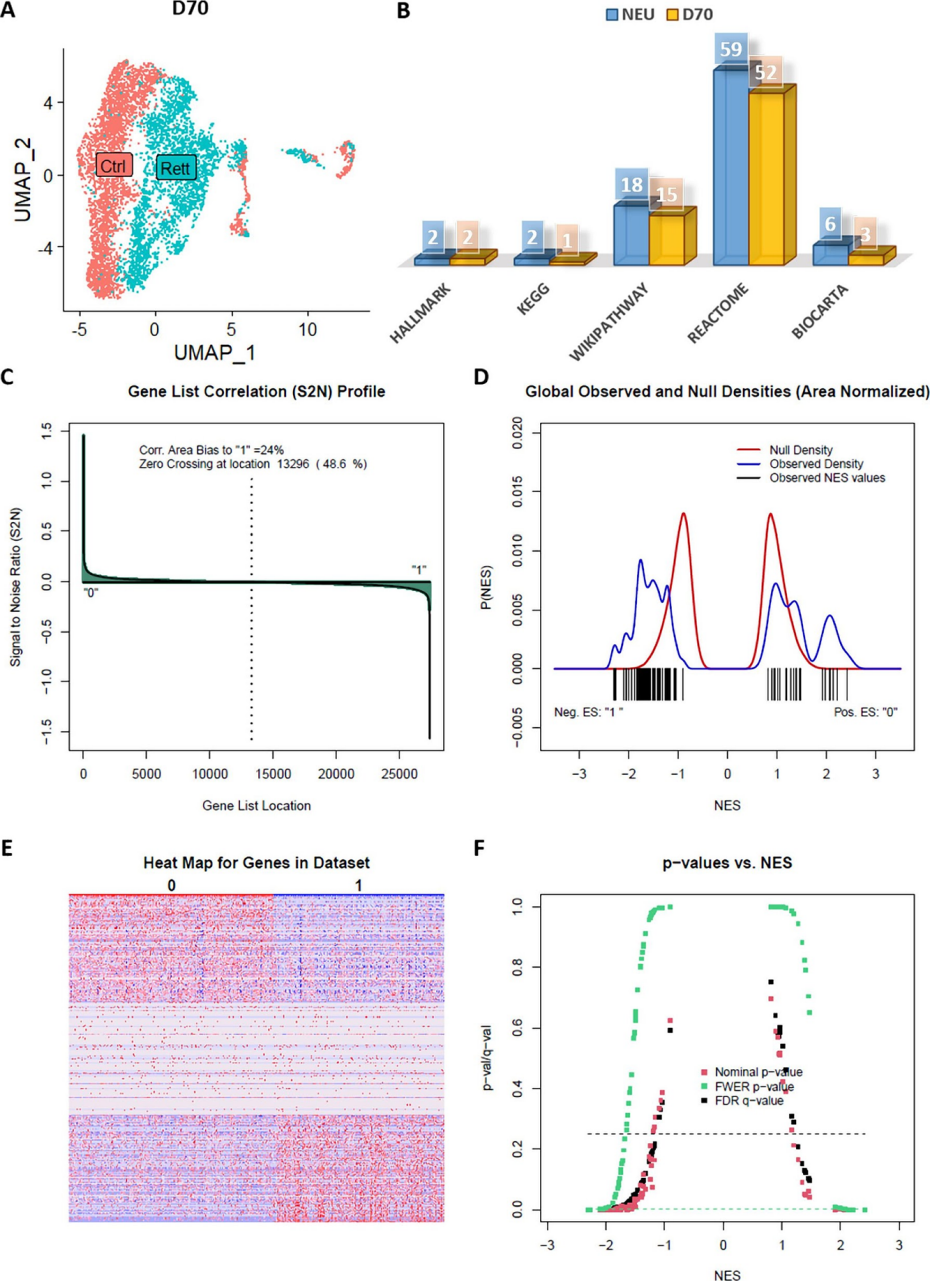
**Gene set enrichment analysis of D70 cortex+ganglionic eminence fused organoid. (A)** Data from D70 fused organoids in terms of phenotype. (**B)** Comparison of significant pathways between NEU and the neuron group from D70 fused organoids. (**C, E)** Correlation between genes and phenotypes. “0” indicates CTRL, “1” indicates RTT. (**D, F)** Number of enriched gene sets that are significant and their densities in terms of phenotype.

There are no transcriptome data that could provide heterogeneity in brain tissue for the GSEA validation of the PM group. Therefore, we used single-cell rna-seq data to validate the GSEA results of the only NEU group. In the study published by Samarasinghe et al., fused cerebral cortex and ganglionic eminence organoids were produced from IPSCs differentiated from fibroblasts of a female patient with 705delG MECP2 mutation.^37^ Because female patients with RTT are heterozygous for the MECP2 mutation, MECP2 expression status in fibroblasts is mosaic^38^. In other words, approximately half of the cells express the nonmutant allele. The ability to obtain both mutant cells and healthy control groups from the same patient eliminates a possible batch effect in the analyses. Cell clusters in the data consist of two main groups: progenitor cells and neurons. We selected groups of neurons by processing D70 fused organoids single-cell rna-seq data. We examined 87 pathways, which we previously determined for NEU with the bulk rna-seq data, in single-cell data and 73 out of 87 pathways in D70 data were determined significant (Figure 6 and Data sets S5).

## Discussion

In this study, we aimed to determine the driver genes and hallmarks of the development of RTT in different sample types. We compared our DEG analysis results with the genes related to RTT and RTT-like syndromes reported in the literature and observed that several previously reported genes such as **MECP2**, **FOXG1**, **CACNA1G**, **SATB2**, **GABBR2**, **MEF2C**, **KCNJ10**, and **CUX2**(39) were also identified in our analysis. In addition, we have identified F8A3, CNTN6, RPE65, and COL19A1 not previously reported in the literature to have differential expression levels in three sample types in RTT. Although the exact function of F8A3 is not known, it has been linked to autism and various cancer types, especially in neuroblastoma.^40,41^ CNTN6, which is involved in sensor-motor neuronal pathways in neurodevelopmental diseases, is reported in the gene list associated with RTT-like syndromes together with KCNJ10.^42,43^ Apart from these findings, we determined that F8A3 and CNTN6 generally displayed the same GO terms as MECP2. Therefore, they are likely to be affected by the MECP2 mutation. Considering the impairment of visual capability observed in RTT, RPE65, which plays a role in retinal development and visual cycle, is worth investigating.^44,45^ However, the fact that RPE65 has no common GO term with MECP2 suggests that it may be effective in RTT independently of MECP2.

CNTN6 encodes a protein that functions as a cell adhesion molecule on the presynaptic membrane and may play a key role as an axon connector in the developing nervous system. Its systematic downregulation across various sample types in RTT tissues as compared to healthy controls can be a therapeutic target by rescuing its expression to normal levels. Although existing databases for available drug candidates did not provide a possible agent, further investigations are advised to investigate its potential in therapeutics. Another disregulated gene, COL19A1, can potentially be targeted to rescue its expression since it is known to play important roles in synaptic developments in various brain regions.^46^ A potential drug called niclosamide has been linked to the increased expression levels of COL19A1^47,48^ and therefore this can be further investigated for the RTT.

One common pattern of functional enrichments from differentially expressed genes was collagen synthesis and extracellular matrix formations. Supporting this, deteriorations in several collagen types in RTT patient-derived cells have been previously reported.^49^ In addition, extracellular matrix structures such as perineuronal nets (PNNs), which cover neuronal cell bodies, are more abundant in RTT as compared to healthy brains.^50,51^ This could be due to disrupted gene expressions of collagens and downregulated COL19A1. The regulatory role of MECP2 on genes involved in processes such as neuronal development, differentiation and signal transduction has been clearly seen.^52−55^ PNNs of parvalbumin-positive (PV+) cells are particularly effective in neuronal cell differentiation.^56^ Differentiation in the OTX2 gene located in the PNNs of PV+ cells could be another factor affecting PNN dysregulation in RTT.

In the WGCNA results, we observed MECP2, F8A3, and CNTN6 in NEU module 3, whereas MECP2 and F8A3 were observed in NPC module 6. The detection of F8A3 and CNTN6 in the same module as MECP2 strengthens the idea of their possible involvement in RTT. Apart from these, the HOX group shows the same behavior in both NPC module 6 and NEU module 3, pointing out the connection of **MECP2, TNRC6A, and HOXA5**. TNRC6A has been implicated in the regulation of dendritic growth and identified in cancer developmental stages and in glioblastoma together with MECP2.^57,58^ In addition, TNRC6A is involved in the uptake of miRNAs through RNA-induced silencing complex, which plays a role in brain development and function.^59^

Since MECP2 and TNRC6A interact in our networks, it is also considered to be worth investigating in RTT studies. The HOX group, which is crucial in embryonic development, cell differentiation, and various diseases,^60,61^ is mostly downregulated in our data. In addition to the presence of a few common GO terms with MECP2,^62^ TNRC6A, HOXA5 connection we detected suggests that the HOX group has a critical role in RTT pathology. UNC5D, which is one of the netrin receptors, is involved in the development of the neuronal system.^63^ The fact that netrin receptors were reported to be effective in neurodevelopmental diseases such as RTT^64^ and that we detected UNC5D as downregulated in our networks supports the idea that UNC5D may affect RTT pathology. When we compared the transcription factors that MECP2 connects in NPC module 6 and NEU module 3, we observed that the genes between the two groups are completely different.

Although NEU module 3 consists of far fewer genes than NPC module 6, MECP2 has connections with 7 transcription factors in NEU, 3 transcriptions in NPC. In addition, the increases in early neural progenitor expression and reductions in neuronal markers with MECP2 deficiency are suggestive of impaired neurogenesis in RTT.^65^ Based on these findings, it can be interpreted that the effect of MECP2 is higher at the level of NEUs than at the level of neuronal progenitor cells.^66^ Moreover, in NEU module 6, ligand-gated ion channel receptors (ionotropic receptors), which are important elements of the central nervous system,^67^ were pointed out. In a study, MECP2-/y mice from birth to observe the effect of RTT on neuronal activity development reported that the GABA developmental shift disappeared and the ratio of glutamatergic/GABAergic postsynaptic currents increased.^68^ In addition, the relationship between MECP2 and GABRA1 receptor was demonstrated in experiments made from cell lines, mouse model, and post-mortem tissue.^69^ The fact that NEU module 6 is specific to the GABRA genes indicates that these genes are involved in the underlying neuronal system disorders observed in RTT.

We applied GSEA to our samples (iPSCs, NPCs, NEUs, and PMs) in order to better understand the development of the RTT mechanism in humans. Considering the significant pathways for each sample type, we observed a consistent increase from iPSCs to NEUs. The number of pathways belonging to the PMs was less than that of NEUs. We attribute this to the cellular diversity and complexity of PMs consisting of brain tissue data, while NEUs consist of only a single type of cell population. Moreover, NEUs were determined as the sample types profoundly displaying the effects of MECP2. According to the arguments presented above, the GSEA results confirmed our hypothesis. The reason that we could not detect any pathways belonging to the iPSC group might be associated with the absence of an effect of disease in the early stage of development. In addition to MAP kinase, SRC kinase, and TGFβ signaling pathways, the prion protein (PRNP) pathway, whose function has not been fully determined, was also detected in NPCs.

PRNP, which has been associated with Alzheimer’s disease, has also been reported in intercellular signal transmission and interaction between signaling pathways.^70,71^ Thus, although the major effect of RTT has not been observed for the NPC group pathways, it may still have a role in the early stages of RTT by deterioration of cellular processes. Significant pathway counts of 87, which we elicited in NEUs compared to those of 8 in NPCs and 0 in IPSCs, confirm the dominant effect of MECP2 on neurons. MECP2 is previously associated with the centrosome and reported to have an effect on cilium production and structure.^72,73^ It is worth mentioning that the cilium acts as sensors in cells and is effective in many cellular functions. At the same time, their control center is the centrosome, as they have a microtubule structure.^74^ In our study, seven genes (**HDAC6**, **C2CD3**, **TRIP11**, **DCTN1**, **TUBA1C**, **DDX5**, and **COX6C**) belonging to seven pathways from “Related with Cilia” were detected in the NEU module 3 network, where MECP2 and HDAC6 are directly related. This finding supports the hypothesis that ciliopathy and RTT are related diseases,^75^ thus a treatment for ciliopathy can be used in RTT. A recent study on MECP2 from mouse olfactory epithelial nuclei has reported that MECP2 can bind directly to the nucleosome, and this binding is stronger with modified histone3 (H3K27me3). Moreover, it was stated that MECP2 competes with other histones in binding to the nucleosome due to its transcriptional regulator role.^76^ Furthermore, it has been shown that MECP2 has a connection with c-Ski and N-CoR repressors besides the mSin3 group (Sin3A, Sin3B) for its transcriptional repressor function.^77^ In line with this information, MECP2 has been shown to exert effects on the chromatin structure as well as its regulatory and suppressive role in gene expression.^78^ In addition, we detected **TNRC6A**, **CREB1**, **PRMT**, **TBL1XR1**, and **SIN3B** which take part in NEU module 3, belonging to three pathways from the “Related with MECP2” subgroup. The fact that TNRC6A has a direct link with genes such as MECP2, CREB1, SIN3B and HOXA5 suggests that it may be one of the active genes in the processes mentioned above. Moreover, we examined the selected pathways in the PM group in three subgroups. In studies conducted to examine the impairments in the immune response processes in RTT, MECP2 is reported to affect neuroimmune homeostasis by creating disturbances in the balance between pro/anti-inflammation. Therefore, irregularities are observed in immune activation.^79,80^ These studies have shown that MECP2 has an important role in regulating monocytes, macrophages, and CD4+ T and B cells.

The 12 pathways we detected in the “Infections, Diseases, and Immune System” subgroup support these studies. In a recent study, peripheral blood of MECP2-null mice was examined, where the complement and coagulation system was shown to be disrupted in the case of RTT.^81^ The pathways of the subgroup “Complement & Coagulation” we detected show that these systems are also disrupted in humans. The “Neuronal Development & Systems” subgroup consists of five pathways. The Netrin-1 signaling pathway is effective in neuronal migration and navigation in the developmental processes of the nervous system, where DCC and UNC5 receptors are active elements of the pathway.^82^ In our results, we previously stated that UNC5D is elicited in the NPC module 6 and NEU module 3 networks. The detection of the active genes of the Netrin-1 signaling pathway in both NPC and NEU networks confirms that the neuronal system connections are significantly affected by MECP2 in the RTT state.^83^ In addition, MECP2 and BDNF deficiencies in RTT have been shown to affect the GABA pathway in which synapse receptors are regulated.^84^ In our data, GABRA1/2/3/4, GABRB1, GABRG3 and GNAL, which are the genes that make up the GABA receptor pathway detected in the PMs, are also observed in our NEU module 6 network. We only validated the NEU group since there are no data for post-mortem tissue validation. As a result of the GSEA we performed with single-cell RNA-seq data, almost all of the NEU pathways we determined in Figure 4B were identified in the validation so our NEU GSEA results with single-cell and bulk RNA-seq data support each other.

When we evaluate our results in general according to DEG analysis, F8A3, CNTN6, and RPE65 are the prominent genes that are worth investigating in future studies. In WGCNA results, we detected a specific module for GABA receptors in NEUs and found the connection of the HOX group with TNRC6A, MECP2 in both NPCs and NEUs that show these structures emerged in RTT. We predicate the inability of detecting significant modules for the iPSC group while constructing the correlation networks to the unbalanced sample counts in the comparison of CTRL iPSC and RTT iPSC. We attribute the inability to detect significant modules for the PM group to the fact that the data from two different studies have heterogeneity such as cingulate cortex, temporal cortex, and frontal lobe, apart from the batch effect due to the integrated use of the data from two different studies. This is also one of the reasons the number of significantly enriched pathways of PMs was lower than that of the NEUs. However, despite this limitation, we were able to detect many biological pathways for changes in RTT pathology as a result of GSEA. In addition to MECP2-centered pathways, the prominence of deterioration of the cilia structure and development at the NEU level shows the effect of mutations in MECP2, especially on neuronal systems. This study shows us changes in RTT pathology across different sample types of humans using transcriptomics analyses in order to determine new possible driver genes and pathways. In addition, the results of this study include focal points for future research and clinical studies.

## Materials and Methods

### Bulk RNA-seq Data Set Preparation and Preanalysis

All data sets were obtained from NCBI Gene Expression Omnibus (GEO; https://www.ncbi.nlm.nih.gov/geo/). The term “Rett Syndrome” was searched in NCBI and 169 BioProjects were identified under the Genome section. By selecting “Human” as the organism groups and “Transcriptome” as the data type, 23 studies out of 169 remained. Among these 23 studies, when we examined the Bulk RNA-seq data with MECP2 mutation and produced with high-throughput sequencing technology, we selected three studies (GSE128380, GSE123753, and GSE107399) containing IPSC, neuronal cells, and post-mortem brain tissue data that can be analyzed together. To obtain all data sets, a snakemake pipeline that uses SRR numbers of all samples as input of the code, kallisto as an aligner, R package“ tximport” (v1.26.1)^85^ for raw count matrix and R package “sleuth” (v0.30.0)^86^ for generating TPM (transcript per million) matrix is prepared. While generating the TPM matrix, only protein-coded genes were chosen from Ensemble by R package “biomaRt” (v2.54.1).^87^ Average values of each row of the TPM matrix were calculated, and the rows with average value ≤1.0 were filtered. Then, log2 transformation was applied to the TPM matrix, and the distribution across samples was examined by using box plots. Coefficient of variation (CV) values of each gene were computed and 2000 genes with the highest CV values were elicited for the PCA. After the boxplot analysis and the PCA, the batch effect observed in the samples was removed by using the R package “SVA” (v3.46.0) and ComBat() function.^88^ A sample-to-sample heatmap was created by using the top 2000 genes from the ComBat-applied/CV-sorted TPM matrix before DEG analysis.

### Differentially Expressed Gene (DEG) Analysis

The technical replication in three tissue (iPSC, NPC, and NEU) samples’ expression values was merged in the row count matrix for this analysis. In order for the number of genes in the analysis not to be excessive, the count matrix was converted to the CPM matrix and sorted according to the CV values of the genes, and the top 10,000 genes were determined for analysis. The selected 10000 genes were taken from the count matrix and analyzed with the R package “DESeq2” (v1.38.3).^21^ After the analysis, up and downregulated genes of four tissue types were shown on volcano plots (Log2FoldChange value ≥1, adjust *p*-value ≤0.05). After that, overlapping differentially expressed genes across all tissue types were identified in Rstudio. To investigate the overlapped genes, DEG lists were given to R package “Clusterprofiler” and gseGO() function^89^ function as an input in Rstudio. Potential drug and molecular interaction information was obtained from Drug Gene Budger (https://maayanlab.cloud/DGB/) for overlapped genes. The potential drugs were elicited based on “cell line” and “log2 Fold Change” parameters (Data sets S6). The DEGs in each sample type were uploaded to STRINGdb (www.string-db.org) in order to obtain gene networks. To get hub genes from the DEGs, a Cytoscape software application called “cytoHubba” is used. We chose the maximal clique centrality (MCC) method and identified the top 15 hub genes. The pathway information on the hub genes is obtained from STRINGdb.

### Weighted Gene Correlation Network Analysis (WGCNA)

Signed coexpression networks for NPC and NEU were built using the R package “WGCNA” (v1.71). To determine coexpression similarity, a threshold power has to be set with the “pickSoftThreshold” function (Figure S4). After determining the soft-threshold value, genes showing similar behavior were separated by using the “blockwise” function to be in the same modules. A table containing the statistical values of the modules was created using the limma:lmfit()/ebayes() functions,^90^ and we selected the modules that were statistically significant (adjust *P* value <0.05). The genes in each significant module were uploaded to STRINGdb (www.string-db.org) in order to get PPI network and enrichment characteristics. The network file belonging to the relevant module was taken from STRINGdb and visualized in the Cytoscape software with ‘Edge-weighted Spring Embedded’ layout.^91^ The HPA (www.proteinatlas.org) database was used to obtain gene-disease and genes-function relations. The shapes of the nodes in the network were adjusted according to the classes (function) of the genes, the node color was adjusted according to the log2FoldChange values, and the node frame color was adjusted according to the disease associations. We used the cytoscape application “cytoHubba” to get hub genes of the modules. We chose the MCC method and identified the top 10 hub genes.

### Gene Set Enrichment Analysis (GSEA)

We prepared a.gct file containing expression information and a.cls file containing samples phenotype information to be used as input for the analysis. Gene sets from Hallmark, Biocarta, Reactome, Wikipathway and Kegg databases were taken from GSEA Molecular Signatures Database (MSigDB, http://www.gsea-msigdb.org/gsea/msigdb/genesets.jsp). The FDR value in the analysis was set to a maximum of 0.25 to get significant enriched pathways.

### Validation of GSEA

Single cell RNA-seq data were obtained from the NCBI (GSE165577). Each sample in the data was processed using the Cell Ranger 4.0.0 pipeline with the provided annotation refdata-gex-GRCh38–2020-A (10× Genomics). The data were combined into a Seurat object and we filtered the cells with number of features (genes) less than 500 or higher than 3 standard deviation more of an average cell and the percentage of mitochondrial content more than 10%. The data were normalized and scaled with the default parameters using Seurat functions NormalizeData(), FindVariableFeatures(), and ScaleData (datExpr, split.by = ‘orig.ident’, do.center = FALSE). For the integration and batch correction, the Seurat-wrapper functions were used. The data were then clustered using FindNeighbors (datExpr, reduction = “iNMF”, dims = 1:20) and FindClusters (datExpr, resolution = 0.3). Cluster marker genes were determined by using the function FindAllMarkers (object = datExpr). Cluster assignments were manually performed with the determined cell-type marker genes from previously published studies. We create a subset by selecting excitatory neurons (upper layer callosal projection neurons (CPNs) and deep layer corticofugal projection neurons (CFuPNs)) and cajal-retzius from seurat_object and take expression matrix from the subset for GSEA validation. We set the significant pathways of NEU as input gene-sets for GSEA. We prepared a.gct and.cls file from the subset seurat_object. The FDR value in the analysis was set to max. of 0.25 to get significant enriched pathways.

## Data Availability

We used Bulk and Single cell RNA-seq data from NCBI. Bulk RNA-seq Data: GSE17399, GSE128753, GSE128380. Single cell RNA-seq Data: GSE165577. Custom code: GitHub (https://github.com/Odabasi-Yusuf/Rett-Syndrome).

## Supporting Information Available

Pre-analysis and quality control of the raw data; gene ontology analysis of differential expressed genes; hub genes of NPC and NEU modules by the MCC method; soft threshold powers of iPSC, NPC, NEU, and PM groups; RNA-seq metadata used in this study; differential gene expression analysis lists; overlapping genes from DEG across the sample types; and WGCNA module table to get the significant ones (PDF)
Data sets S1–S6 including pathways of top 15 hub genes from iPSC, NPC, NEU and PM's DEGs and PPI network of IPSC, NPC, NEU and PM, genes and log2fc values of NPC and NEU modules, GSEA of NPC modules and NEU modules, STRING informations of NPC and NEU modules, GSEA of D70 fused organoids, and drugs and small molecules to downregulate expression of RPE65, upregulate expression of CNTN6 and COL19A1 (XLSX)
