# Supplementary material for: Comprehensive Transcriptomic Investigation of Rett Syndrome Reveals Increasing Complexity Trends from Induced Pluripotent Stem Cells to Neurons with Implications for Enriched Pathways
Source: ACS Omega. 2023 Nov 8;8(46):44148–62. doi: 10.1021/acsomega.3c06448 (PMC10666228; doi:10.1021/acsomega.3c06448)
Supplement: Supplementary file 1 — ao3c06448_si_001.pdf [file ao3c06448_si_001.pdf]

Comprehensive transcriptomic investigation of Rett syndrome reveals increasing complexity trends from iPSC to neurons with implications for enriched pathways

Yusuf Caglar Odabasi, Sena Yanasik, Pelin Saglam-Metiner, Yasin Kaymaz*, Ozlem Yesil-Celiktas*

Department of Bioengineering, Faculty of Engineering, Ege University, 35100, Izmir, Turkey

MSc. Yusuf Caglar Odabasi

Department of Bioengineering, Faculty of Engineering, Ege University, 35100, Izmir, Turkey

MSc. Sena Yanasik

Department of Bioengineering, Faculty of Engineering, Ege University, 35100, Izmir, Turkey

PhD. Pelin-Saglam-Metiner

Department of Bioengineering, Faculty of Engineering, Ege University, 35100, Izmir, Turkey

Dr. Yasin Kaymaz*

Department of Bioengineering, Faculty of Engineering, Ege University, 35100, Izmir, Turkey

Prof. Dr. Ozlem Yesil-Celiktas*

Department of Bioengineering, Faculty of Engineering, Ege University, 35100, Izmir, Turkey

***Corresponding authors e-mail:** ozlem.yesil.celiktas@ege.edu.tr, yasin.kaymaz@ege.edu.tr

SUPPLEMENTARY FIGURES

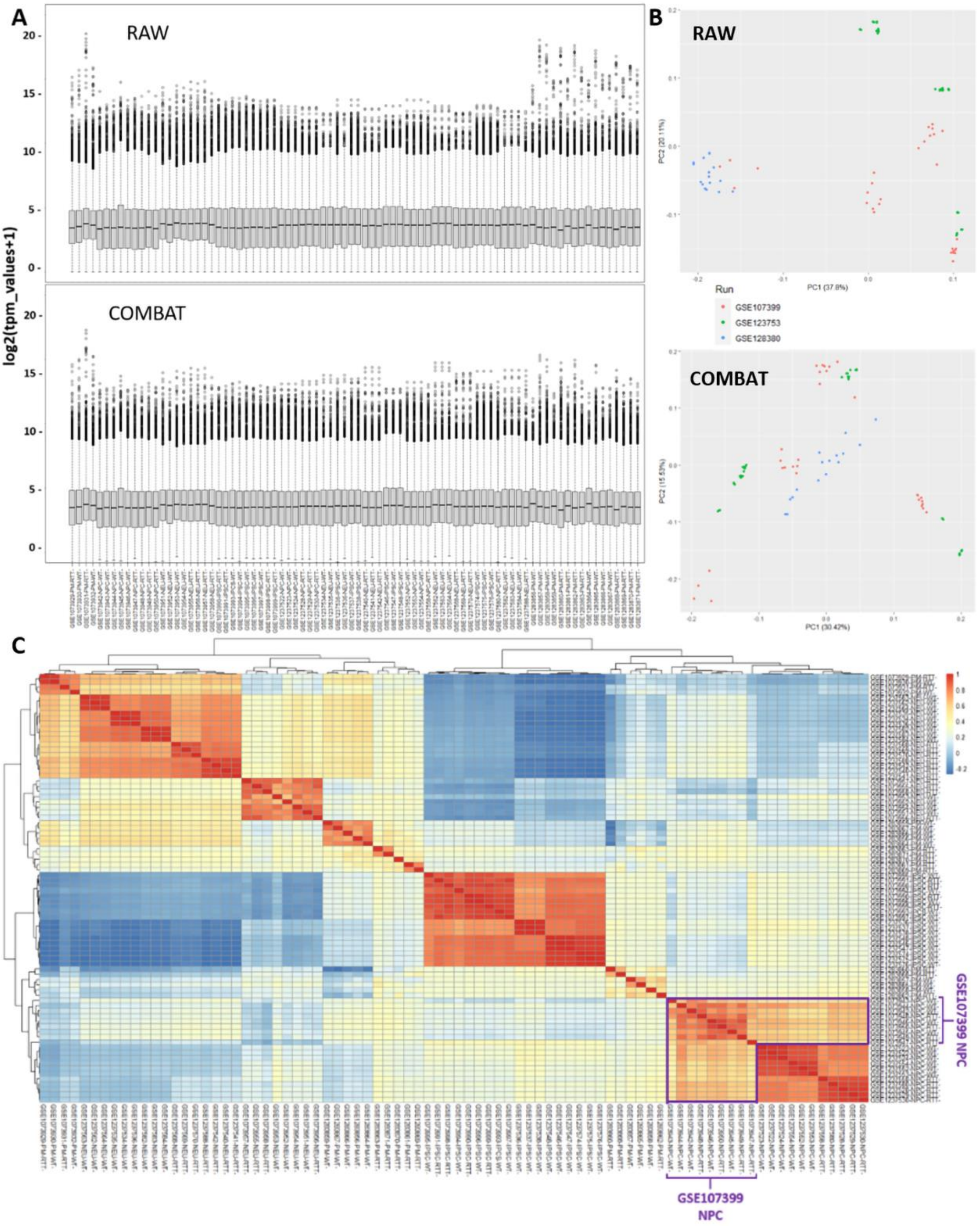

Supplementary Figure 1. Pre-analysis and quality control of the raw data.,

A Batch correction with comparing raw and corrected data set's boxplot. **B** A Batch correction with PCA graphs. Comparing raw and corrected dataset in terms of Run. **C** Sample to sample heatmap of all sample type. TPM matrix which consist of CV-sorted top 2000 genes, is used to get heatmap. GSE107399's NPC (purple line) is extracted from the datasets.

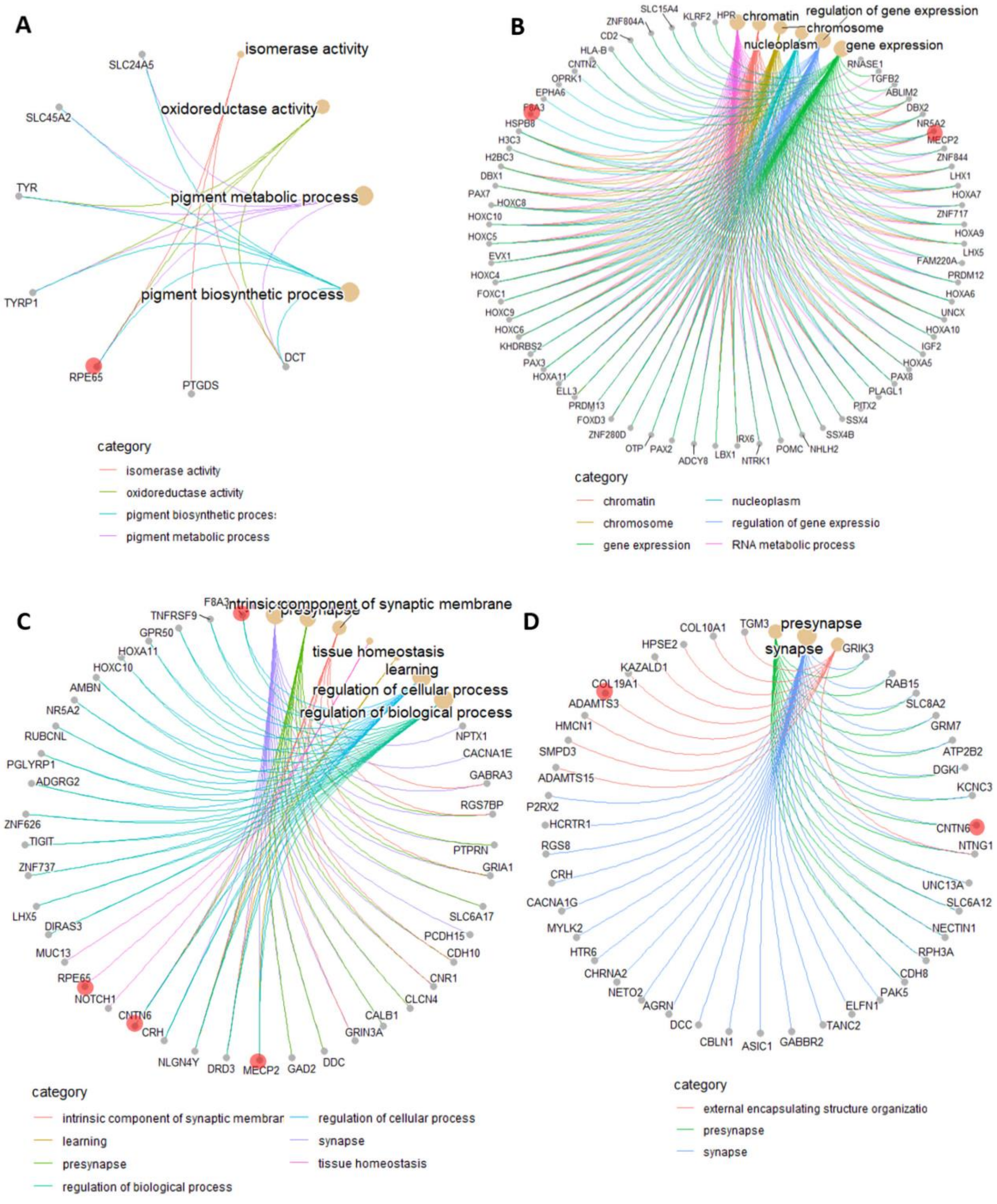

Supplementary Figure 2. Gene ontology analysis of differential expressed genes

A GO terms for IPSC group. In top 3 GO MF and BP terms of differentially expressed genes,RPE65 is in isomerase activity, oxidoreductase activity, pigment metabolic process and pigment biosynthetic process. **B** GO terms for NPC group. In top 3 GO CC and BP terms of differentially expressed genes, F8A3 is in nucleoplasm, MECP2 is in all of 6 GO terms. **C** GO terms for NEU group. In top 3 GO CC and BP terms of differentially expressed genes, CNTN6 is in intrinsic component of synaptic membrane, presynapse, synapse, regulation of cellular process and regulation of biological process. MECP2 is in synapse, regulation of cellular process, regulation of biological process and learning. RPE65 is in tissue homeostasis. F8A3 is in regulation of cellular process and regulation of biological process. **D** GO terms

for PM group. In top 3 GO CC and BP terms of differentially expressed genes, CNTN6 is in presynapse and synapse. COL19A1 is in external encapsulating structure organization.

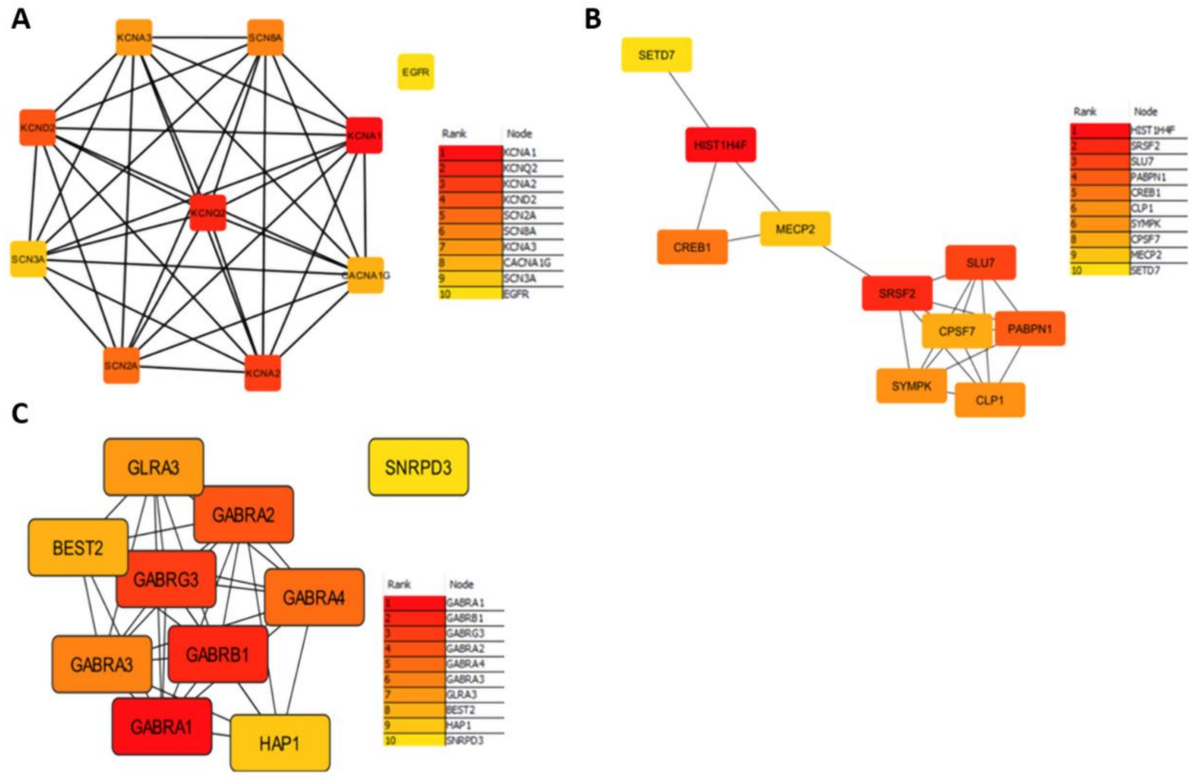

Supplementary Figure 3. Hub genes of NPC and NEU Modules by MCC method.

A Top 10 hub genes of NPC module 6. **B** Top 10 hub genes of NEU module 3. **C** Top 10 hub genes of NEU module 8. **D** Top 10 hub genes of NEU module 6.

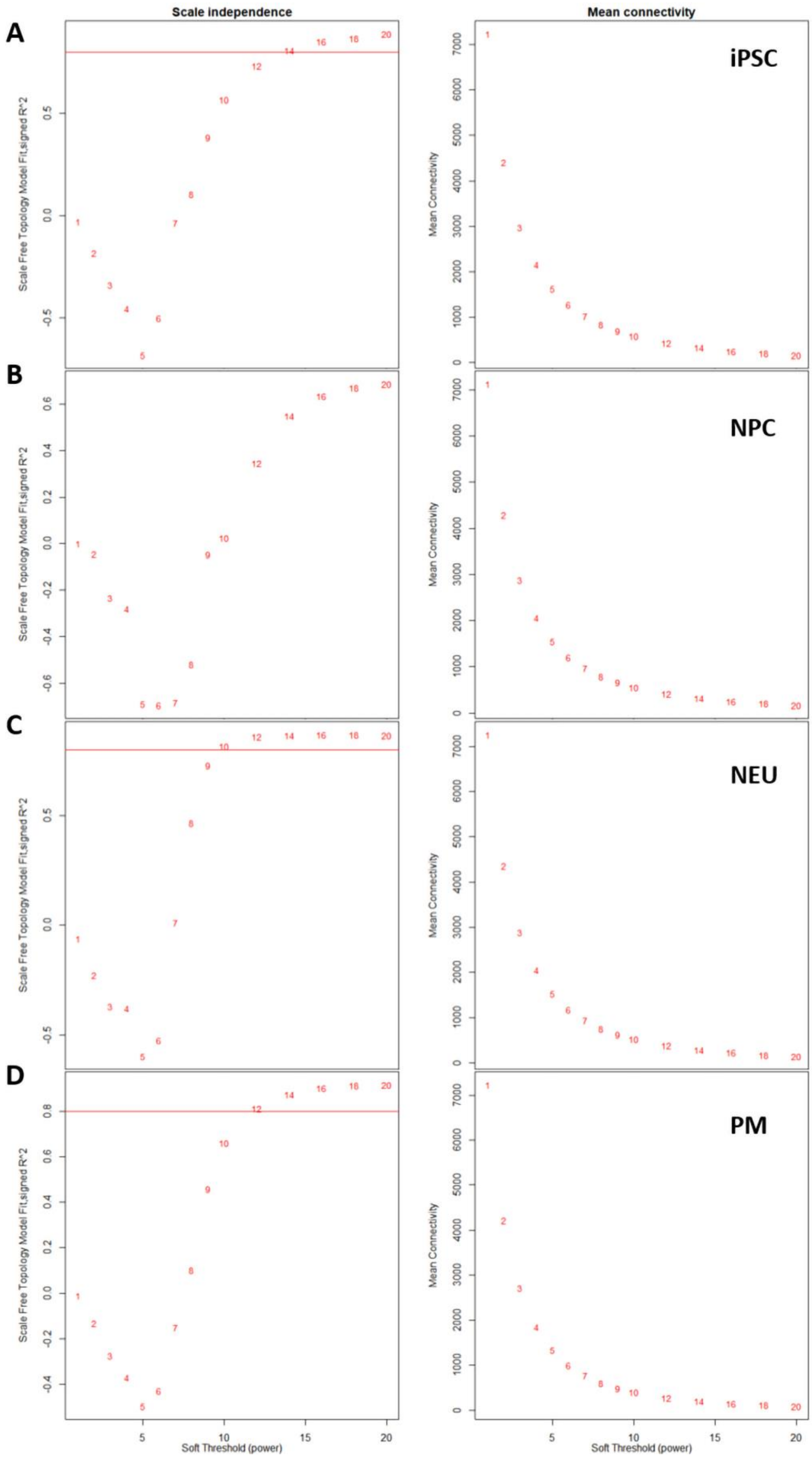

Supplementary Figure 4. Soft threshold Powers of iPSC, NPC, NEU and PM groups. The threshold line is set to 0.8.

A We selected 14 is a power for iPSC group. **B** We didn't reach the 0.8 threshold so we selected 14 as a power for NPC group. **C** We selected 10 is a power for NEU group. **D** We selected 12 as a power for PM group.

SUPPLEMENTARY TABLES

Table S1. RNA-seq metadata used in this study.

Run	Sample	phenotype	mutation	tissue
GSE107399	GSM2866248	RTT	c.378-2 A>G, splice site, g(hg19) ChrX:153296903 A>G	Postmortem
GSE107399	GSM2866249	WT	-	Postmortem
GSE107399	GSM2866250	RTT	NA ?	Postmortem
GSE107399	GSM2866252	WT	-	Postmortem
GSE107399	GSM2866253	WT	-	NPC
GSE107399	GSM2866255	WT	-	NPC
GSE107399	GSM2866256	WT	-	NPC
GSE107399	GSM2866257	WT	-	NPC
GSE107399	GSM2866258	WT	-	NPC
GSE107399	GSM2866259	RTT	1461A>G	NPC
GSE107399	GSM2866261	RTT	1461A>G	NPC
GSE107399	GSM2866262	RTT	705delG	NPC
GSE107399	GSM2866263	RTT	705delG	NPC
GSE107399	GSM2866265	WT	-	NEU
GSE107399	GSM2866266	WT	-	NEU
GSE107399	GSM2866268	WT	-	NEU
GSE107399	GSM2866269	WT	-	NEU
GSE107399	GSM2866270	RTT	1461A>G	NEU
GSE107399	GSM2866272	RTT	1461A>G	NEU
GSE107399	GSM2866273	RTT	705delG	NEU
GSE107399	GSM2866274	RTT	705delG	NEU
GSE107399	GSM2866276	RTT	1461A>G	IPSC
GSE107399	GSM2866277	RTT	1461A>G	IPSC
GSE107399	GSM2866278	WT	-	IPSC
GSE107399	GSM2866279	WT	-	IPSC
GSE107399	GSM2866281	WT	-	IPSC
GSE107399	GSM2866282	WT	-	IPSC
GSE107399	GSM2866283	WT	-	IPSC
GSE107399	GSM2866285	RTT	705delG	IPSC
GSE107399	GSM2866286	RTT	705delG	IPSC
GSE123753	GSM3510817	WT	-	NPC

GSE123753	GSM3510818	WT	-	NPC
GSE123753	GSM3510819	WT	-	NPC
GSE123753	GSM3510823	RTT	MECP2 exons 3-4 deletion	NPC
GSE123753	GSM3510824	RTT	MECP2 exons 3-4 deletion	NPC
GSE123753	GSM3510825	RTT	MECP2 exons 3-4 deletion	NPC
GSE123753	GSM3510829	WT	-	NEU
GSE123753	GSM3510830	WT	-	NEU
GSE123753	GSM3510811	WT	-	IPSC
GSE123753	GSM3510831	WT	-	NEU
GSE123753	GSM3510812	WT	-	IPSC
GSE123753	GSM3510813	WT	-	IPSC
GSE123753	GSM3510835	RTT	MECP2 exons 3-4 deletion	NEU
GSE123753	GSM3510836	RTT	MECP2 exons 3-4 deletion	NEU
GSE123753	GSM3510837	RTT	MECP2 exons 3-4 deletion	NEU
GSE123753	GSM3510841	WT	-	IPSC
GSE123753	GSM3510842	WT	-	IPSC
GSE123753	GSM3510843	WT	-	IPSC
GSE123753	GSM3510847	WT	-	NPC
GSE123753	GSM3510848	WT	-	NPC
GSE123753	GSM3510849	WT	-	NPC
GSE123753	GSM3510853	RTT	MECP2 exons 3-4 deletion	NPC
GSE123753	GSM3510857	WT	-	NEU
GSE123753	GSM3510858	WT	-	NEU
GSE123753	GSM3510859	WT	-	NEU
GSE123753	GSM3510863	RTT	MECP2 exons 3-4 deletion	NEU
GSE123753	GSM3510864	RTT	MECP2 exons 3-4 deletion	NEU
GSE123753	GSM3510865	RTT	MECP2 exons 3-4 deletion	NEU
GSE123753	GSM3510869	WT	-	IPSC
GSE123753	GSM3510870	WT	-	IPSC
GSE123753	GSM3510871	WT	-	IPSC
GSE123753	GSM3510875	RTT	MECP2 exons 3-4 deletion	NPC
GSE123753	GSM3510877	WT	-	NEU
GSE123753	GSM3510878	WT	-	NEU
GSE123753	GSM3510879	WT	-	NEU

GSE123753	GSM3510883	RTT	MECP2 exons 3-4 deletion	NEU
GSE128380	GSM3673208	WT	-	Postmortem
GSE128380	GSM3673209	WT	-	Postmortem
GSE128380	GSM3673210	WT	-	Postmortem
GSE128380	GSM3673211	WT	-	Postmortem
GSE128380	GSM3673212	RTT	No mutation in MECP2 reported	Postmortem
GSE128380	GSM3673213	RTT	c.473 C > T (cDNA) // p.Thr158Met (protein)	Postmortem
GSE128380	GSM3673214	RTT	Exon Del	Postmortem
GSE128380	GSM3673215	RTT	No mutation in MECP2 reported	Postmortem
GSE128380	GSM3673216	WT	-	Postmortem
GSE128380	GSM3673217	WT	-	Postmortem
GSE128380	GSM3673218	WT	-	Postmortem
GSE128380	GSM3673219	WT	-	Postmortem
GSE128380	GSM3673220	RTT	No mutation in MECP2 reported	Postmortem
GSE128380	GSM3673221	RTT	c.473 C > T (cDNA) // p.Thr158Met (protein)	Postmortem
GSE128380	GSM3673222	RTT	Exon Del	Postmortem
GSE128380	GSM3673223	RTT	No mutation in MECP2 reported	Postmortem
GSE165577	GSM5044261	WT	-	hPSC-dervied brain organoids - Cortex and MGE // D56
GSE165577	GSM5044261	WT	-	hPSC-dervied brain organoids - Cortex and MGE // D56
GSE165577	GSM5044262	RTT	705delG	hPSC-dervied brain organoids - Cortex and MGE // D56
GSE165577	GSM5044262	RTT	705delG	hPSC-dervied brain organoids - Cortex and MGE // D56
GSE165577	GSM5044263	WT	-	hPSC-dervied brain organoids - Cortex-MGE fusions // D70
GSE165577	GSM5044263	WT	-	hPSC-dervied brain organoids - Cortex-MGE fusions // D70
GSE165577	GSM5044264	RTT	705delG	hPSC-dervied brain organoids - Cortex-MGE fusions // D70
GSE165577	GSM5044264	RTT	705delG	hPSC-dervied brain organoids - Cortex-MGE fusions // D70
GSE165577	GSM5044265	WT	-	hPSC-dervied brain organoids - Cortex-MGE fusions // D100

GSE165577	GSM5044265	WT	-	hPSC-dervied brain organoids - Cortex-MGE fusions // D100
GSE165577	GSM5044265	WT	-	hPSC-dervied brain organoids - Cortex-MGE fusions // D100
GSE165577	GSM5044265	WT	-	hPSC-dervied brain organoids - Cortex-MGE fusions // D100
GSE165577	GSM5044266	RTT	705delG	hPSC-dervied brain organoids - Cortex-MGE fusions // D100
GSE165577	GSM5044266	RTT	705delG	hPSC-dervied brain organoids - Cortex-MGE fusions // D100
GSE165577	GSM5044266	RTT	705delG	hPSC-dervied brain organoids - Cortex-MGE fusions // D100
GSE165577	GSM5044266	RTT	705delG	hPSC-dervied brain organoids - Cortex-MGE fusions // D100

Table S2. Differential gene expression analysis lists.

IPSC_UP	IPSC_DOWN	NPC_UP	NPC_DOWN	NEU_UP	NEU_DOWN	PM_UP	PM_DOWN
1 "NEUROD6"	1 "F8A3"	1 "ZXDA"	1 "KLRF2"	1 "HSFX1"	1 "F8A3"	1 "ADH4"	1 "GPR21"
2 "DCT"	2 "FAM24B"	2 "CAT"	2 "RGPD2"	2 "NOTCH1"	2 "CNTN6"	2 "SERPINA3"	2 "GSTM1"
3 "OC90"	3 "MAGEA2"	3 "TAFA5"	3 "TMEM132D"	3 "CEACAM5"	3 "TNFRSF9"	3 "IFI30"	3 "NPIPB2"
4 "TYRP1"	4 "H1-1"	4 "FOXA2"	4 "CLEC2A"	4 "RPE65"	4 "CRH"	4 "HLA-DRB5"	4 "P2RX2"
5 "PAX6"	5 "C9orf64"	5 "C21orf91"	5 "DBX1"	5 "MUC13"	5 "GPR50"	5 "CP"	5 "CYP1A1"
6 "TYR"	6 "ZNF492"	6 "OTX2"	6 "PAX7"	6 "NUTM2F"	6 "HOXA11"	6 "SERPINA5"	6 "ZNF511-PRAP1"
7 "PTGDS"	7 "TRIM61"	7 "TUSC1"	7 "HOXC8"	7 "CDX2"	7 "HOXC10"	7 "MUC12"	7 "SERPINB2"
8 "DAPL1"	8 "CXCL5"	8 "GDF1"	8 "HOXC10"	8 "ACE2"	8 "AMBN"	8 "PRSS33"	8 "CYP24A1"
9 "CNTN5"	9 "GABRA2"	9 "NKX2-1"	9 "SLC15A4"	9 "S100A14"	9 "NLGN4Y"	9 "C4B_2"	9 "CALML3"
10 "CRYGC"	10 "ZNF229"	10 "DCAF12L2"	10 "HOXC5"	10 "DUOX2"	10 "NR5A2"	10 "SELE"	10 "SPON2"
11 "SLC45A2"	11 "MRO"	11 "ZNF248"	11 "LCN9"	11 "MYO3B"	11 "DRD3"	11 "CHI3L2"	11 "PRTN3"
12 "RPE65"	12 "TRIM4"	12 "CHODL"	12 "EVX1"	12 "CRYBA1"	12 "MECP2"	12 "NPC1L1"	12 "KRT78"
13 "MAB21L1"	13 "GCNT4"	13 "RRAGB"	13 "HOXC4"	13 "IGFL2"	13 "PNMA6F"	13 "MCEMP1"	13 "MYB"
14 "MAL"	14 "IKZF1"	14 "NLRP2"	14 "FOXC1"	14 "FMOD"	14 "RUBCNL"	14 "RNASE2"	14 "DRGX"

15 "CRYAA"	15 "ALG6"	15 "TBC1D26"	15 "HOXC9"	15 "GATA6"	15 "PGLYRP1"	15 "C7orf61"	15 "TGM3"
16 "CRYAB"	16 "NRG3"	16 "ZNF680"	16 "CNTN6"	16 "DSG3"	16 "ADGRG2"	16 "HBD"	16 "PRAP1"
17 "PRPH"		17 "PKIB"	17 "LRRC61"	17 "RAB17"	17 "ZNF626"	17 "EVC2"	17 "HCRTR1"
18 "CLEC4F"		18 "TRPC6"	18 "HOXC6"	18 "TM4SF1"	18 "TIGIT"	18 "S100A8"	18 "FREM3"
19 "TRPM1"		19 "SLC39A12"	19 "KHDRBS2"	19 "IGFBP7"	19 "ZNF737"	19 "ADGRE1"	19 "PROKR2"
20 "RELN"		20 "FOXG1"	20 "GLT1D1"	20 "AGR2"	20 "LHX5"	20 "TNFRSF6B"	20 "C4orf54"
21 "TTR"		21 "ZNF705A"	21 "TNNT2"	21 "DPP4"	21 "DIRAS3"	21 "CCL2"	21 "RTP1"
22 "TFEC"		22 "TAF11L11"	22 "UNC5D"	22 "NCAM2"	22 "PRMT8"	22 "HSPB1"	22 "CACNA1S"
23 "ATP6V1B1"		23 "MBD3L2"	23 "PAX3"	23 "MPZL2"	23 "LGR5"	23 "KRT15"	23 "HES5"
24 "MAB21L2"		24 "LHFPL3"	24 "ADAMTS8"	24 "COX11"	24 "H2BC14"	24 "MKNK2"	24 "COL10A1"
25 "GRIA2"		25 "NKX3-2"	25 "HOXA11"	25 "GJB2"	25 "GAD2"	25 "HBG2"	25 "FBLN7"
26 "ITGB6"		26 "PTPRT"	26 "ELL3"	26 "CASP4"	26 "ZNF717"	26 "HSPA1A"	26 "PTH2R"
27 "PDK4"		27 "SCEL"	27 "PRDM13"	27 "CAPG"	27 "DDC"	27 "S100A9"	27 "ATP6V0A4"
28 "NR2E1"		28 "MBD3L3"	28 "OPRK1"	28 "PGF"	28 "GRIN3A"	28 "HSPA1B"	28 "LIME1"
29 "LCNL1"		29 "H3Y1"	29 "FOXD3"	29 "COL1A1"	29 "ZNF844"	29 "CA1"	29 "SMIM24"
30 "SIX3"		30 "TFAP2A"	30 "H3C3"	30 "SIM1"	30 "COL19A1"	30 "FOXJ1"	30 "ADRA1D"
31 "SLC24A5"		31 "TRIM43"	31 "MYBPH"	31 "PCDHGA10"	31 "ABCB1"	31 "S100A12"	31 "RGS8"
32 "CNTN3"		32 "RAX"	32 "CYP4F22"	32 "CYP2W1"	32 "CALB1"	32 "SCGB1D2"	32 "GPR78"
33 "TRIM63"		33 "ZNF558"	33 "ZNF280D"	33 "GOLT1A"	33 "CLCN4"	33 "BAG3"	33 "HPSE2"
34 "WT1"		34 "LMX1A"	34 "ZNF804A"	34 "SLC2A12"	34 "GPR83"	34 "ZFP36"	34 "CRH"
35 "BARHL2"		35 "KCNQ3"	35 "IGFN1"	35 "BNC1"	35 "SLCO1A2"	35 "IL1RN"	35 "KAZALD1"
36 "ZIC4"		36 "TAF11L12"	36 "OTP"	36 "EMCN"	36 "CNR1"	36 "MLKL"	36 "NEK2"
37 "KRT17"		37 "PNPO"	37 "TRIM6-TRIM34"	37 "SH3TC1"	37 "SPATA6L"	37 "IL1RL1"	37 "NUTM2B"
38 "PALMD"		38 "GPR12"	38 "EGFL6"	38 "SEMA3C"	38 "CDH10"	38 "ANKRD22"	38 "CA4"
39 "TMEM176A"		39 "TCEAL5"	39 "SMOC1"	39 "FHL2"	39 "LEFTY2"	39 "SBSN"	39 "CYP26B1"
40 "CFB"		40 "PRAMEF12"	40 "PAX2"	40 "TUBA1C"	40 "CDH9"	40 "CXCL1"	40 "CALHM1"
41 "ERICH5"		41 "EFEMP1"	41 "ADCY8"	41 "SOWAHA"	41 "CPNE9"	41 "S100A3"	41 "GPR45"
42 "TRIM67"		42 "IL1RAPL2"	42 "LBX1"	42 "ACOT9"	42 "PCDH15"	42 "SFN"	42 "PTPN3"
43 "NDST4"		43 "MAFIP"	43 "IRX6"	43 "CNN2"	43 "AMT"	43 "NQO1"	43 "NCR3LG1"
44 "GCGR"		44 "ZIC4"	44 "NTRK1"		44 "RTL5"	44 "C4A"	44 "NWD2"
45 "LRP2"		45 "LHX2"	45 "PRMT8"		45 "C11orf87"	45 "CXCL10"	45 "TNFRSF18"

46 "DACH1"		46 "MALRD1"	46 "CD2"		46 "CHRDL1"	46 "ALAS2"	46 "CDH8"
47 "LMX1A"		47 "SLC13A5"	47 "ECHDC3"		47 "SAMD3"	47 "SERPINA1"	47 "ASCL2"
48 "GPRASP1"		48 "CA12"	48 "POMC"		48 "RTL1"	48 "VSIG4"	48 "FBN3"
49 "PTPRQ"		49 "FEZF1"	49 "ZNF511-PRAP1"		49 "SLC6A17"	49 "SLC4A1"	49 "COL19A1"
50 "SLC4A5"		50 "ERMN"	50 "SLC6A5"		50 "SATB2"	50 "FCN3"	50 "L3MBTL1"
51 "DLK1"		51 "NFIA"	51 "NHLH2"		51 "DARS2"	51 "FPR1"	51 "ADAM19"
52 "POU4F2"		52 "BCAN"	52 "SSX4"		52 "SEZ6L"	52 "HBB"	52 "KCNV1"
53 "PDZRN4"		53 "TM4SF1"	53 "SSX4B"		53 "GRIA1"	53 "LILRA5"	53 "CACNA1G"
54 "TEKT1"		54 "FOXL2"	54 "PLAC9"		54 "PTPRN"	54 "ICAM1"	54 "IL12RB2"
55 "GPNMB"		55 "SYNDIG1"	55 "PITX2"		55 "EN1"	55 "PLA1A"	55 "MYLK2"
56 "BIRC7"		56 "FOLH1"	56 "MFSD4A"		56 "C2orf73"	56 "FCGBP"	56 "SETBP1"
57 "PRSS56"		57 "SHISA6"	57 "HPR"		57 "DMGDH"	57 "GMPR"	57 "CATSPERZ"
58 "CAVIN2"		58 "NRK"	58 "HLA-B"		58 "ANKRD30A"	58 "TMPRSS3"	58 "MAPK15"
59 "NR4A3"		59 "S100B"	59 "H2BC3"		59 "MARCHF11"	59 "ITGB4"	59 "NBPF3"
60 "TRH"		60 "TBX2"	60 "PLAGL1"		60 "RGS7BP"	60 "DNAJB1"	60 "LRRC55"
61 "NEUROG2"		61 "IFI44"	61 "ROBO3"		61 "GABRA3"	61 "HMOX1"	61 "HTR6"
62 "COL1A2"		62 "KCNA3"	62 "PAX8"		62 "PRKG2"	62 "CA12"	62 "PCDHA8"
63 "SOX6"		63 "MKX"	63 "SLC22A15"		63 "SLC7A3"	63 "ITGB3"	63 "ANO3"
64 "DCDC1"		64 "MEOX2"	64 "HOXA5"		64 "NRIP3"	64 "MICB"	64 "CHRNA2"
65 "CNGB3"		65 "NFIX"	65 "IGF2"		65 "GPR158"	65 "BCL2A1"	65 "SPEF1"
66 "COL8A1"		66 "ACOT2"	66 "HOXA10"		66 "CACNA1E"	66 "C10orf105"	66 "RASGRF1"
67 "MEIS1"		67 "RPE65"	67 "CNTN2"		67 "ZNF619"	67 "SLC1A5"	67 "ADAMTS3"
68 "NR2F2"		68 "SLC8A3"	68 "RUBCNL"		68 "NPTX1"	68 "FKBP5"	68 "COL26A1"
69 "CXCL14"		69 "ANGPT1"	69 "UNCX"		69 "C4orf33"	69 "MPZL2"	69 "RPH3A"
70 "RLBP1"		70 "RGS6"	70 "HOXA6"			70 "MAB21L2"	70 "INF2"
71 "NSG2"		71 "ALDH1L1"	71 "PRDM12"			71 "OTOS"	71 "DUSP6"
72 "PRSS33"		72 "COL1A2"	72 "CASQ2"			72 "PDLIM1"	72 "SSTR1"
73 "MITF"		73 "CACNG5"	73 "FRAS1"			73 "TNFRSF11B"	73 "FCHO1"
74 "LHX2"		74 "KCNA2"	74 "EPHA6"			74 "RTL1"	74 "NECTIN1"
75 "ZIC1"		75 "CBLN2"	75 "C10orf105"			75 "CD14"	75 "SLC6A12"
76 "ZNF503"		76 "DPF3"	76 "ALKAL1"			76 "RAC2"	76 "SLC36A1"
77 "DIRAS2"		77 "NXPH1"	77 "FAM220A"			77 "PFN4"	77 "MARCHF4"
78 "AGT"		78 "SYNPR"	78 "SCUBE2"			78 "SYTL1"	78 "C5orf34"
79 "ABCA4"		79 "PRDM16"	79 "F8A3"			79 "CISH"	79 "UNC13A"

80 "SLC30A8"		80 "PRRX1"	80 "WNT7B"			80 "HBA1"	80 "PLXNA1"
81 "ALDH1A1"		81 "CXCL14"	81 "LHX5"			81 "CD44"	81 "PRKCD"
82 "SERPINE3"		82 "KCNE5"	82 "HOXA9"			82 "FAM167B"	82 "FRAS1"
83 "DES"		83 "GLUD2"	83 "CBR1"			83 "NUPR1"	83 "FBXO9"
84 "RRAD"		84 "GATM"	84 "ZNF717"			84 "TSLP"	84 "NTNG1"
85 "WFIKKN2"		85 "TLE2"	85 "HSPB8"			85 "TYMP"	85 "NETO2"
86 "MEIS2"		86 "SCN1A"	86 "HOXA7"			86 "HS3ST3B1"	86 "KCNB2"
87 "PENK"		87 "ASTN1"	87 "LHX1"			87 "CHI3L1"	87 "NFASC"
88 "NPFFR1"		88 "KCND3"	88 "ZNF844"			88 "CFI"	88 "DLK2"
89 "IFITM10"		89 "SLC6A11"	89 "DAAM2"			89 "TFCP2L1"	89 "SLC6A7"
90 "CELF6"		90 "GASK1B"	90 "SHISA3"			90 "SECTM1"	90 "MYSM1"
91 "STMN4"		91 "FBXO25"	91 "TMEM229A"			91 "RLN2"	91 "HMCN1"
92 "LIMCH1"		92 "CMKLR1"	92 "NLGN4Y"			92 "AQP1"	92 "HUNK"
93 "FRZB"		93 "MASP1"	93 "MECP2"			93 "HGD"	93 "KCNH5"
94 "TNFRSF14"		94 "MLC1"	94 "PPP1R1A"			94 "CRYAB"	94 "CHAD"
95 "ERCC5"		95 "MYL9"	95 "NR5A2"			95 "OXTR"	95 "CNTN6"
96 "IFI44L"		96 "DGKG"	96 "PLXDC1"			96 "HBA2"	96 "SMPD3"
97 "RTN4RL1"		97 "NCAM2"	97 "DSP"			97 "MTHFD2"	97 "ADAMTS15"
98 "WNT11"		98 "PMP2"	98 "DBX2"			98 "SLC14A1"	98 "TMEM38A"
99 "WNT2B"		99 "ADGRL4"	99 "LAYN"			99 "SLC16A9"	99 "GPR149"
100 "LRRTM2"		100 "ZNF439"	100 "AGTR1"			100 "MS4A6A"	100 "SPRN"
101 "PLCL1"		101 "NFIB"	101 "ABLIM2"			101 "TIMP1"	101 "KCNC3"
102 "HSF4"		102 "ZNF562"	102 "DIO3"			102 "ID3"	102 "AGRN"
103 "RARB"		103 "TGFBI"	103 "RARRES2"			103 "TRIM5"	103 "FAT2"
104 "LY6H"		104 "CASQ1"	104 "TGFB2"			104 "RGR"	104 "DGKI"
105 "CDH7"		105 "ATP1A2"	105 "LRFN2"			105 "IL18R1"	105 "TPBGL"
106 "SOSTDC1"		106 "GABBR2"	106 "MYLPF"			106 "IL32"	106 "DNAH5"
107 "HSD17B2"		107 "SRPX2"	107 "ABCC4"			107 "HPR"	107 "EGR1"
108 "KCNA4"		108 "SCN3A"	108 "RNASE1"			108 "SDSL"	108 "SAMD3"
109 "NR1H4"		109 "EYA4"	109 "HLA-C"			109 "NR5A2"	109 "E2F1"
110 "ENPP2"		110 "CHRM3"	110 "ATF7-NPFF"			110 "MDK"	110 "DCC"
111 "UBC"		111 "VAX2"	111 "DHRS3"			111 "FXYD3"	111 "LINGO3"
112 "PDGFRA"		112 "BMP5"	112 "ABCB1"			112 "IL17RB"	112 "HMGCR"
113 "COL5A1"		113 "FLI1"	113 "OTOL1"			113 "RBM47"	113 "TMEM179"
114 "TSHZ1"		114 "EHD2"	114 "USP44"			114 "IFITM3"	114 "KCNT1"

115 "UBAP1L"		115 "ABCA8"	115 "ADM"			115 "LIMS2"	115 "LPA"
116 "TIMP3"		116 "TM4SF18"	116 "RHOU"			116 "IFITM2"	116 "TMEM121B"
117 "COL1A1"		117 "DDIT4L"	117 "ANOS1"			117 "TAGLN"	117 "NIPAL2"
118 "COL2A1"		118 "IL1RAP"	118 "RSPO3"			118 "PI16"	118 "CBLN1"
119 "HSBP1L1"		119 "CD69"	119 "CNTN5"			119 "H2AC6"	119 "KCNK4"
120 "CABP7"		120 "PCDHGA10"	120 "CNTN3"			120 "GPRC5A"	120 "NCALD"
121 "TGFB2"		121 "C1QL4"	121 "PTGES"			121 "C1R"	121 "KIF21B"
122 "RGS9BP"		122 "RHOJ"	122 "XKRX"			122 "PLP2"	122 "TONSL"
123 "BRINP3"		123 "DCDC2"	123 "SFRP2"			123 "SPR"	
124 "VSX2"		124 "BMP2"	124 "WFDC1"			124 "VIM"	
125 "FOXP2"		125 "METTL7A"	125 "NDNF"			125 "DIO3"	
126 "HOXB5"		126 "KCNJ6"	126 "DENND2D"			126 "HVCN1"	
127 "COLGALT2"		127 "GRM3"	127 "SLC27A2"			127 "STEAP4"	
128 "FOXG1"		128 "KCNN4"	128 "NEUROG2"			128 "IRF1"	
129 "NPNT"		129 "CALB1"	129 "GRIA3"			129 "MSX2"	
130 "CCDC187"		130 "EMX2"	130 "STAC"			130 "VSIR"	
131 "BCO1"		131 "SEL1L3"	131 "MSC"			131 "TEAD4"	
132 "PLCH2"		132 "NALF1"	132 "SPTSSB"			132 "BST2"	
133 "PCDH9"		133 "ADGRB1"	133 "STARD5"			133 "CD163"	
134 "DCLK2"		134 "TNC"	134 "TMEM42"			134 "FZD7"	
135 "ADAMTS18"		135 "SLFN12"	135 "HSFX2"			135 "A4GALT"	
136 "SGIP1"		136 "IGFBP7"	136 "CPVL"			136 "SLC44A3"	
137 "EPHA5"		137 "RANBP3L"	137 "PADI2"			137 "AEBP1"	
138 "CLIP4"		138 "OSMR"	138 "SEMA6D"			138 "LAIR1"	
139 "TGM2"		139 "ABCB4"	139 "MYOG"			139 "SQOR"	
140 "KCNA5"		140 "SLC6A1"	140 "SBF2"			140 "IL1R1"	
141 "ELN"		141 "PCYT1B"	141 "ERVMER34-1"			141 "RBPMS"	
142 "CPLX3"		142 "HSFX1"	142 "RCAN2"			142 "MT1M"	
143 "P2RX2"		143 "COL9A3"	143 "LEF1"			143 "C21orf62"	
144 "SEMA3E"		144 "SCUBE1"	144 "HS3ST3B1"			144 "SYTL4"	
145 "BEST1"		145 "SOX18"	145 "RBM20"			145 "TMEM176B"	
146 "KANK4"		146 "KCND2"	146 "ATP8A1"			146 "LPIN3"	
147 "RBM20"		147 "LYPD6B"	147 "ZNF626"			147 "LGALS3"	
148 "TMEM176B"		148 "GRID2"	148 "CNNM1"			148 "MT1X"	

149 "OCA2"		149 "POSTN"	149 "TGFBR3"			149 "HSPB8"	
150 "CRX"		150 "IL1RAPL1"	150 "OLFM3"			150 "ANXA2"	
151 "PRDM16"		151 "DKK1"	151 "TPD52"			151 "SH2D7"	
152 "CDH12"		152 "SYTL5"	152 "SV2C"			152 "PLIN2"	
153 "DCN"		153 "A2M"	153 "HS3ST3A1"			153 "IGFBP3"	
154 "SLIT1"		154 "KHDRBS3"	154 "TAC3"			154 "BCL2L12"	
155 "SYNM"		155 "ETS1"	155 "ESRRB"			155 "CSF1"	
156 "CLSTN2"		156 "ZNF37A"	156 "GALNTL6"			156 "AHNAK"	
157 "OTX1"		157 "GNA14"	157 "NKX6-2"			157 "TENT5C"	
158 "C7"		158 "SLIT1"	158 "H2BC14"			158 "ACSL5"	
159 "TRPM3"		159 "ASCL1"	159 "COL19A1"			159 "BATF2"	
160 "TMEM235"		160 "DNAJC22"	160 "ROR1"			160 "FGR"	
161 "MYH14"		161 "B3GALT5"	161 "STEAP4"			161 "ACTA2"	
162 "COL11A1"		162 "TMEM271"	162 "GPR39"			162 "PIR"	
163 "DIO2"		163 "KCNE4"	163 "GOLGA6L4"			163 "PAX6"	
164 "OR51E2"		164 "PLPPR5"	164 "FAAH"			164 "TLR3"	
165 "TBX15"		165 "GPER1"	165 "PALMD"			165 "GADD45G"	
166 "MYH3"		166 "SYNC"	166 "OTULINL"			166 "OSMR"	
167 "MYOF"		167 "ITGB4"	167 "ZNF619"			167 "OSGIN1"	
168 "EPAS1"		168 "TMEM71"	168 "TKTL1"			168 "CXCL5"	
169 "LSAMP"		169 "POU5F1B"	169 "DOCK10"			169 "GMNN"	
170 "CUBN"		170 "OCIAD2"	170 "SGSM1"			170 "UPP1"	
171 "ARL4D"		171 "GRIK4"	171 "CDK18"			171 "ADRB2"	
172 "HRC"		172 "ZNF239"	172 "COL11A1"			172 "MSX1"	
173 "ZNF536"		173 "BNC2"	173 "PCSK6"			173 "RP1"	
174 "LTBP3"		174 "LMO1"	174 "KCNMB2"			174 "SELL"	
175 "CD302"		175 "RAB27B"	175 "CNTNAP2"			175 "CEBPD"	
176 "COL8A2"		176 "CACNG8"	176 "ADAMTS12"			176 "KIAA0040"	
177 "FBN1"		177 "ITPRID2"	177 "KCNK12"			177 "ACADL"	
178 "C4A"		178 "GRAMD2B"	178 "NEUROG1"			178 "TNFAIP3"	
179 "AKAP6"		179 "PRPH"	179 "CCDC3"			179 "DHRS3"	
180 "TSHZ2"		180 "ENPEP"	180 "SPOCK3"			180 "GEM"	
181 "MEGF9"		181 "C21orf62"	181 "ABHD3"			181 "SMIM1"	
182 "ZNF593OS"		182 "ADAMTS3"	182 "PDZRN4"			182 "HGF"	

183 "TMEM86A"		183 "RUNX2"	183 "H1-0"			183 "PDLIM4"	
184 "MYRF"		184 "SCG2"	184 "STAT4"			184 "LY96"	
185 "CHRNA3"		185 "GREM1"	185 "HOXA4"			185 "APOL6"	
186 "AKAP12"		186 "SLFN11"	186 "HS6ST3"			186 "GLP1R"	
187 "GIPR"		187 "PLAT"	187 "H2AC14"			187 "OASL"	
188 "TPPP"		188 "KCNK10"	188 "PERP"			188 "RHOBTB3"	
189 "SORCS2"		189 "PCP4"	189 "AMT"			189 "TMBIM1"	
190 "SNAP91"		190 "PCDHB5"	190 "GRM2"			190 "GRAMD2B"	
191 "PLD5"		191 "ACOT9"	191 "CNTNAP3"			191 "TCF7"	
192 "CNTNAP1"		192 "IFI44L"	192 "MAL2"			192 "STK17B"	
193 "CCP110"		193 "PLPP2"	193 "CTSH"			193 "AOC3"	
194 "FLRT1"		194 "LAMB1"	194 "NEFM"			194 "PYGL"	
195 "MAPT"		195 "KCNJ10"	195 "ZNF486"			195 "PLAAT4"	
196 "SSC5D"		196 "PLXNB3"	196 "PNMA6A"			196 "SCIN"	
197 "STARD9"		197 "ANKFN1"	197 "KCNK2"			197 "ANGPT1"	
		198 "RARG"	198 "KCNN2"			198 "HEBP2"	
		199 "CTNNA3"	199 "CMTM8"			199 "ZNF44"	
		200 "KLF8"	200 "FOXB1"			200 "HLA-DRA"	
		201 "RAI2"	201 "DSC2"			201 "C1S"	
		202 "ZNF578"	202 "KIAA0319"			202 "HTR1D"	
		203 "GRIK5"	203 "SORL1"			203 "SLC7A2"	
		204 "FXYD5"	204 "GRM8"			204 "GNA14"	
		205 "PEG3"	205 "NEBL"			205 "CD99"	
		206 "IGSF11"	206 "STMN2"			206 "TRIM47"	
		207 "TXLNB"	207 "CARMIL1"			207 "GADD45A"	
		208 "ZNF835"	208 "PLXNA2"			208 "HLA-DRB1"	
		209 "SPON1"	209 "RAB11FIP4"			209 "CFAP53"	
		210 "THY1"	210 "KLHL20"			210 "GBP1"	
		211 "NFATC1"	211 "STON1-GTF2A1L"			211 "F2R"	
		212 "PCDHGB1"	212 "ZIC3"			212 "PRKD2"	
		213 "FEZF2"	213 "CEP85L"			213 "BCAR3"	
		214 "EMP1"	214 "TSPAN15"			214 "SP110"	
		215 "CAPG"	215 "CHRNB4"			215 "LCTL"	
		216 "ARHGAP31"	216 "SLCO1A2"			216 "TIFA"	

		217 "TUBB8"	217 "CPNE8"			217 "TGFB3"	
		218 "STC1"	218 "ADAM11"			218 "ANG"	
		219 "TMEM108"	219 "CHRD"			219 "NMUR2"	
		220 "RORB"	220 "HS3ST5"			220 "H4C15"	
		221 "ARSJ"	221 "ZNF737"			221 "SRPX"	
		222 "ABCC9"	222 "MYO3A"			222 "TNFAIP8"	
		223 "CHST2"	223 "WFIKKN1"			223 "LIPG"	
		224 "ZIC1"	224 "FAM131C"			224 "FTL"	
		225 "CCDC80"	225 "KLHL32"			225 "IFITM1"	
		226 "S100A11"	226 "SLC6A15"			226 "GPR35"	
		227 "DPPA2"	227 "SALL4"			227 "PNP"	
		228 "DNAH6"	228 "DSG2"			228 "HMGB2"	
		229 "ERBB3"	229 "SAMD12"			229 "LIX1"	
		230 "PDLIM3"	230 "PLXDC2"			230 "GLIS3"	
		231 "PCDHA11"	231 "ZNF506"			231 "TNIP2"	
		232 "TFCP2"	232 "HOOK1"			232 "LYZ"	
		233 "PLP2"	233 "MBP"			233 "SHISA6"	
		234 "PDE4D"	234 "ADCY2"			234 "ANGPTL1"	
		235 "PRTFDC1"	235 "GFRA1"			235 "NMI"	
		236 "ITGA3"	236 "FBXW4"			236 "FLNA"	
		237 "DUSP26"	237 "MGMT"			237 "PALLD"	
		238 "CAVIN1"	238 "PID1"			238 "THNSL2"	
		239 "PDGFRB"	239 "SKAP2"			239 "PHEX"	
		240 "SHH"	240 "TAF4B"			240 "HACD4"	
		241 "TTN"	241 "ALPL"			241 "ACTL6A"	
		242 "ID3"	242 "CPLX2"			242 "PPP1R3D"	
		243 "SCARF1"	243 "KCNB2"			243 "HSPD1"	
		244 "SNTB1"	244 "NEFL"			244 "TRIM61"	
		245 "MCHR1"	245 "MYO1D"			245 "RFLNB"	
		246 "RAPGEF4"	246 "HHIP"			246 "APOBEC3G"	
		247 "FGF12"	247 "PRR5"			247 "ADAMTS2"	
		248 "MAF"	248 "EFNA5"			248 "FXYD5"	
		249 "PPARGC1A"	249 "DACT1"			249 "CSRP2"	
		250 "DOK5"	250 "DBH"			250 "HFE"	

		251 "THBS2"	251 "FAM149A"			251 "PLOD1"	
		252 "EFHC2"	252 "SLC7A3"			252 "DTX3L"	
		253 "S100A10"	253 "NELL2"			253 "ZC3HAV1"	
		254 "MDGA2"	254 "CDH23"			254 "PLBD1"	
		255 "IKBKE"	255 "DPYSL3"			255 "LONRF3"	
		256 "F8A1"	256 "RGS10"			256 "ZNF844"	
		257 "SLC17A6"	257 "ST8SIA3"			257 "MSN"	
		258 "SLC9A6"	258 "GLI1"			258 "CLU"	
		259 "LAMA4"	259 "SPATA18"			259 "PCBD1"	
		260 "ANKRD44"	260 "NRIP3"			260 "MAP7D3"	
		261 "AGAP2"	261 "DSC3"			261 "LTBP1"	
		262 "EGFR"	262 "CTTNBP2"			262 "UNG"	
		263 "JPH4"	263 "CD55"			263 "BNIP2"	
		264 "TANC1"	264 "RORA"			264 "SIGLEC10"	
		265 "INHBA"	265 "THEMIS2"			265 "RPGR"	
		266 "NFIC"	266 "RPL39L"			266 "ERBB2"	
		267 "PCDHB8"	267 "PATJ"			267 "IL15"	
		268 "FREM2"	268 "CUX2"			268 "CMYA5"	
		269 "ELMOD1"	269 "SNX10"			269 "CPS1"	
		270 "CASP8"	270 "NALF2"			270 "ARL17A"	
		271 "FAM167A"	271 "DDB2"			271 "RNASE4"	
		272 "AFF2"	272 "ZNF544"			272 "CEBPB"	
		273 "FAM110C"	273 "PRICKLE1"				
		274 "CRABP2"	274 "ACKR3"				
		275 "BFSP1"	275 "SYTL4"				
		276 "DPPA4"	276 "ENPP4"				
		277 "HEY2"	277 "ZNF525"				
		278 "SORCS1"	278 "LDLRAP1"				
		279 "C2orf72"	279 "STC2"				
		280 "MAGI2"	280 "EXPH5"				
		281 "ACTG2"	281 "PODN"				
		282 "CDH8"	282 "RIOX2"				
		283 "BHLHE40"	283 "LRRC1"				
		284 "ID1"	284 "TMEM51"				
		285 "NLGN3"	285 "FZD9"				
		286 "ELK3"	286 "FRRS1L"				
		287 "FXYD7"	287 "CNTNAP3B"				

		288 "MATN2"	288 "HMCN1"				
		289 "ITPR2"	289 "CRYBG3"				
		290 "PCDHGA4"	290 "GDF7"				
		291 "TENM1"	291 "HIVEP2"				
		292 "SLC6A20"	292 "ASS1"				
		293 "PCDHGB4"	293 "CORO2A"				
		294 "CTSO"	294 "FHAD1"				
		295 "TFPI"	295 "STAMBPL1"				
		296 "PAQR6"	296 "TNFRSF1B"				
		297 "SOSTDC1"	297 "SALL3"				
		298 "ZNF436"	298 "ZNF385D"				
		299 "RASGRP2"	299 "ZNF536"				
		300 "ZDHHC15"	300 "RNF175"				
		301 "SLC4A4"	301 "ZNF135"				
		302 "THSD1"	302 "ZNF845"				
		303 "CACNB2"	303 "SHISAL1"				
		304 "NDST3"	304 "P4HA2"				
		305 "LRRC7"	305 "ADD2"				
		306 "CACNG4"	306 "CPS1"				
		307 "PHYHIPL"	307 "HSPA12A"				
		308 "MEF2C"	308 "SCRT2"				
		309 "TIE1"	309 "PRSS23"				
		310 "SLC38A3"	310 "RETREG1"				
		311 "RASD2"	311 "GCNT4"				
		312 "TMIGD2"	312 "DCN"				
		313 "EPHB1"	313 "COL3A1"				
		314 "PLCB4"	314 "CLVS2"				
		315 "GRIN2D"	315 "KCNJ11"				
		316 "MPPED2"	316 "GAS1"				
		317 "EN2"	317 "VAT1L"				
		318 "CD248"	318 "CHRDL1"				
		319 "ENOX1"	319 "RGS4"				
		320 "INKA1"	320 "MGAT4C"				
		321 "PTPRZ1"	321 "ALK"				
		322 "KCNJ9"	322 "NFASC"				
		323 "LRRC17"	323 "NR2F1"				
		324 "SLC5A12"	324 "ADAMTSL1"				
		325 "CNPY1"	325 "CREG1"				
		326 "FAM43A"	326 "CFAP91"				

		327 "PLAU"	327 "ANKRD63"				
		328 "HRK"	328 "ADAMTS16"				
		329 "SRD5A1"	329 "SLC2A1"				
		330 "DLL4"	330 "SFRP1"				
		331 "BCL11B"	331 "PYGL"				
		332 "SOX14"	332 "IGDCC3"				
		333 "PCDHGB7"	333 "PPP1R16B"				
		334 "WDR49"	334 "SIRPA"				
		335 "SEZ6"	335 "B3GAT2"				
		336 "MYT1L"	336 "STX3"				
		337 "TMPRSS5"	337 "FAT4"				
		338 "SHANK2"	338 "KIZ"				
		339 "LYL1"	339 "TMEM178A"				
		340 "ATP1B2"	340 "ANKRD18B"				
		341 "SOX6"	341 "DNAH11"				
		342 "SYT17"	342 "PTPN18"				
		343 "EGR1"	343 "ACTR3B"				
		344 "ZCCHC24"	344 "IRAG1"				
		345 "ZNF300"	345 "NAALAD2"				
		346 "TMBIM1"	346 "ADCY5"				
		347 "EDNRB"	347 "DTWD1"				
		348 "SLC6A9"	348 "NRXN3"				
		349 "KCTD12"	349 "BTN3A2"				
		350 "RIN1"	350 "PAIP2B"				
		351 "SLC1A3"	351 "RASEF"				
		352 "S100A16"	352 "TSGA10"				
		353 "AHNAK"	353 "FBXO32"				
		354 "ENKUR"	354 "BMPR1B"				
		355 "GAS7"	355 "UBB"				
		356 "RCAN3"	356 "FAM89A"				
		357 "DISP3"	357 "CACNA2D2"				
		358 "SERPINE1"	358 "SP9"				
		359 "SEMA6A"	359 "IGFBP3"				
		360 "LONRF2"	360 "HECW1"				
		361 "B3GALT1"	361 "NMU"				
		362 "FLNC"	362 "LUZP2"				
		363 "RHPN2"	363 "SCRT1"				
		364 "PLEKHH2"	364 "ART5"				

		365 "TMEM121"	365 "PDLIM1"				
		366 "MPP1"	366 "YPEL3"				
		367 "ZNF585A"	367 "SH3GL2"				
		368 "TNS1"	368 "CA2"				
		369 "MMP14"	369 "RIMS4"				
		370 "SULF1"	370 "NRTN"				
		371 "LRRC4C"	371 "ERBB4"				
		372 "GAREM1"	372 "GRIK2"				
		373 "FLRT1"	373 "KIT"				
		374 "NDP"	374 "KATNAL2"				
		375 "ZNF528"	375 "POU2F2"				
		376 "EHHADH"	376 "EDIL3"				
		377 "HTRA1"	377 "TRPS1"				
		378 "F3"	378 "MICA"				
		379 "RGS14"	379 "SYTL2"				
		380 "ZNF605"	380 "ZBTB16"				
		381 "CDKN1A"	381 "FADS3"				
		382 "PPP1R3B"	382 "TIFA"				
		383 "LHX4"	383 "DCC"				
		384 "AHRR"	384 "RIMS1"				
		385 "BHLHE41"	385 "UBASH3B"				
		386 "CNN1"	386 "NRIP1"				
		387 "PCDHB12"	387 "PRTG"				
		388 "ZNF454"	388 "CHRNA3"				
		389 "LRRN2"	389 "DIAPH2"				
		390 "PCDH9"	390 "RPL22L1"				
		391 "DCLK2"	391 "TIAM2"				
		392 "NPAS3"	392 "TSPAN18"				
		393 "CEBPZOS"	393 "GNAL"				
		394 "SHOX2"	394 "RIMS2"				
		395 "ADGRB3"	395 "FGF9"				
		396 "TSPAN12"	396 "ADARB2"				
		397 "SORCS2"	397 "PAK3"				
		398 "GSTA4"	398 "STXBP5"				
		399 "DDX60"	399 "PTCH1"				
		400 "C1QTNF6"	400 "PARP8"				
		401 "KCNF1"	401 "ZNF761"				
		402 "ZNF283"	402 "USP25"				
		403 "SNX13"	403 "CACNA1G"				
		404 "KANK1"	404 "DNM3"				

		405 "FAM172A"	405 "SOX3"				
		406 "GPM6B"	406 "DNER"				
		407 "LGALS1"	407 "REEP6"				
		408 "ATL3"	408 "CTSL"				
		409 "LTBP3"	409 "PAX6"				
		410 "HSPA1A"	410 "OCLN"				
		411 "DHX36"	411 "ZDHHC4"				
		412 "SUMF1"	412 "CDYL"				
		413 "LRRC8B"	413 "HPS1"				
		414 "BMP8A"	414 "SLC38A1"				
		415 "PCDHGA8"	415 "GPC3"				
		416 "TRAF3IP2"	416 "PRKCE"				
		417 "INSYN2B"	417 "REEP1"				
		418 "IFI16"	418 "PAK1"				
		419 "SH3KBP1"	419 "RTTN"				
		420 "ARHGAP42"	420 "PKDCC"				
		421 "PRAG1"	421 "MOCS1"				
		422 "ZNF138"	422 "ARL4D"				
		423 "ADGRA1"	423 "SULT1C4"				
			424 "APBA1"				
			425 "ZNF839"				
			426 "NDUFAF1"				
			427 "PCSK9"				
			428 "FTH1"				
			429 "ZNF354C"				
			430 "IFNGR2"				
			431 "PDE5A"				
			432 "RPS6KA6"				
			433 "SOX1"				
			434 "C5"				
			435 "C3orf18"				
			436 "IGSF9B"				
			437 "TRIP6"				
			438 "AKAP1"				
			439 "KIAA1522"				
			440 "TCEAL7"				

Table S3. Overlapping genes from DEG across the sample types.

IPSC--NPC--UP	IPSC--NEU--UP	IPSC--PM--UP	IPSC--NPC--DOWN	IPSC--NEU--DOWN	NPC--NEU--UP	NPC--PM--UP	NPC--NEU--DOWN	NPC--PM--DOWN	NEU--PM--UP	NEU--PM--DOWN	IPSC--NPC--NEU--UP	IPSC--NPC--NEU--DOWN	NPC--NEU--PM--DOWN
RPE65	RPE65	PAX6	F8A3	F8A3	TM4SF1	CA12	HOXC10	CNTN6	MPZL2	CNTN6	RPE65	F8A3	CNTN6
PRPH	COL1A1	CRYAB	GCNT4		RPE65	SHISA6	CNTN6	ZNF511		CRH			COL19A1
ZIC4		MAB21L2			NCAM2	ANGPT1	HOXA11	PRAP1		COL19A1			
LMX1A		PRSS33			PCDHGA10	OSMR	PRMT8	FRAS1		SAMD3			
COL1A2		TMEM176B			IGFBP7	GNA14	RUBCNL	COL19A1					
SOX6		C4A			HSFX1	ITGB4	F8A3	KCNB2					
CXCL14					ACOT9	GRAMD2B	LHX5	HMCN1					
LHX2					CAPG	C21orf62	ZNF717	NFASC					
ZIC1						FXYD5	ZNF844	DCC					
IFI44L						PLP2	NLGN4Y	CACNA1G					
SOSTDC1						ID3	MECP2						
FOXG1						TMBIM1	NR5A2						
PCDH9						AHNAK	ABCB1						
DCLK2						HSPA1A	ZNF626						
PRDM16							H2BC14						
SLIT1							COL19A1						
LTBP3							ZNF619						
SORCS2							AMT						
FLRT1							SLC01A2						
							ZNF737						
							SLC7A3						
							NRIP3						
							CHRDL1						

Table S4. WGCNA modules tablet o get the significant ones.

**IPSC**				
module	logFC	AveExpr	P.Value	adj.P.Val
ME24	0.3407859331301979	-1,81E-02	0.011106683581612186	0.21764674249114016
ME1	0.29734097963904577	-4,01E-01	0.02661491903437817	0.21764674249114016
ME23	-0.2815662651403485	-2,31E-02	0.03573141713286872	0.21764674249114016
ME2	0.27821429312651713	-1,58E-01	0.03798026719575157	0.21764674249114016
ME20	-0.2660114066792033	-5,71E-02	0.04721361914242829	0.21764674249114016
ME8	-0.26529487154761705	8,85E-02	0.047810108111045725	0.21764674249114016
ME16	0.2564069054092646	2,33E-02	0.055751919688401125	0.21764674249114016
ME29	0.2538162988803649	-2,12E-01	0.05826428100212984	0.21764674249114016
ME22	0.24932955623430328	3,01E-01	0.06283839950710364	0.21764674249114016
ME17	0.24531390255285918	4,93E-01	0.06718099150201898	0.21764674249114016

ME12	-0.2442224019921159	5,40E-03	0.06840326192578691	0.21764674249114016
ME31	-0.23086314965981045	3,15E-04	0.08490148803710877	0.2371465194792468
ME3	0.22744228376100456	2,08E-01	0.08960886673364638	0.2371465194792468
ME11	-0.22379432758169993	-1,33E-01	0.09485860779169872	0.2371465194792468
ME7	-0.18622438695371488	2,39E-01	0.1643973558847443	0.36839698244537206
ME10	-0.18446652839651295	6,55E-02	0.16841004911788438	0.36839698244537206
ME4	0.17267699813276388	2,39E-01	0.19724569454655097	0.4007985268150483
ME9	0.16930471878954692	2,10E-01	0.20612495664773914	0.4007985268150483
ME30	-0.16179207775482032	2,14E-01	0.22694476296949553	0.4180561423122286
ME32	-0.14373612662772223	6,36E-02	0.28301465539772014	0.47170234400964306
ME21	-0.14373410881198254	-5,01E-01	0.2830214064057858	0.47170234400964306
ME28	0.12915436349295178	-6,55E-02	0.3346672727116462	0.5324252065867099
ME25	0.11791137539878137	1,16E-01	0.3784077955896378	0.5758379498103184
ME5	0.10863669933358525	-1,46E-01	0.41702599671894325	0.591658149181199
ME33	0.10733816375164562	4,41E-02	0.42261296370085644	0.591658149181199
ME18	0.06687371092885958	-5,20E-02	0.6172973223820561	0.8309771647450757
ME27	0.052826792166049595	-4,93E-02	0.6930336530881864	0.8983769577069083
ME34	-0.040432722959013026	4,03E-01	0.7625475348916428	0.9148199900697592
ME26	-0.030491793282576216	2,08E-01	0.8197576865053783	0.9148199900697592
ME0	0.02588212844537987	-1,29E+00	0.8466369724465477	0.9148199900697592
ME6	-0.02568628162322592	2,51E-01	0.847783176877828	0.9148199900697592
ME14	0.025400027978279373	5,78E-03	0.8494590724857047	0.9148199900697592
ME19	-0.022151273528938274	-1,04E-01	0.8685251955912526	0.9148199900697592
ME15	0.01873076523026009	-3,24E-03	0.8886822760677662	0.9148199900697592
ME13	0.01277928892372303	2,31E-02	0.9239193742651963	0.9239193742651963
**NPC**				
ME6	0.5920531006941433	-9,84E-02	6,61E+10	0.001852003968038222
ME2	-0.5369869670060704	-3,63E-02	5,89E+11	0.00576708263869895
ME21	-0.5277876790499867	4,04E-01	7,98E+11	0.00576708263869895
ME8	0.5202445467896847	-3,41E-01	0.0010142484015087019	0.00576708263869895
ME4	-0.5197546207328789	-2,14E-01	0.0010298361854819554	0.00576708263869895
ME9	0.5043750695572944	4,10E-03	0.0016327955007296594	0.007619712336738411
ME10	0.429337048762485	1,23E-02	0.010337798831109237	0.04135119532443695
ME5	0.41798993512930815	1,31E-01	0.01304345694656972	0.04565209931299402
ME23	-0.34080277510860113	-1,40E+00	0.04994981110184411	0.15539941231684837
ME7	-0.2886640972680828	2,90E-02	0.10240680252638824	0.2867390470738871
ME14	-0.2807819317295495	-1,03E+00	0.11291478267260721	0.2874194468030002
ME3	0.26712556560786155	1,51E+00	0.1329185671529846	0.296033140843109
ME12	-0.26424152186133487	7,19E-02	0.13744395824858632	0.296033140843109
ME17	-0.2421694511343549	-1,32E-01	0.17570162958289298	0.33816722588056874
ME24	0.23512536663733205	1,02E+00	0.18930039940114202	0.33816722588056874
ME26	0.22756774278565847	-1,74E+00	0.20465873804794074	0.33816722588056874
ME20	-0.22499872964066545	6,31E-05	0.21006231760053873	0.33816722588056874

ME18	0.22158223814774775	-1,80E-01	0.2173932166375085	0.33816722588056874
ME19	-0.19934281923566932	6,15E-03	0.26919216611416763	0.3894696157875341
ME13	0.1957639244705977	5,30E-01	0.2781925827053815	0.3894696157875341
ME22	0.17707162705128374	1,16E+00	0.32819500084841824	0.4375933344645576
ME15	-0.15041499343531872	6,31E-03	0.408053207834301	0.5193404463345649
ME27	0.13933312011421042	9,59E-02	0.44410470726746887	0.5406492088473535
ME16	0.12365117423903564	2,27E-01	0.4978266846295445	0.5583380820175039
ME0	-0.12096710571919729	2,88E+02	0.507326362842176	0.5583380820175039
ME25	0.11685065050490392	-2,59E-01	0.5220624278900344	0.5583380820175039
ME1	0.11235072101860104	-1,02E-01	0.5383974362311645	0.5583380820175039
ME11	0.0795135823427938	-4,54E-01	0.6641246850041863	0.6641246850041863

NEU

ME3	0.332927888903168	1,76E-03	2,05E+09	0.000451225051991966
ME13	0.274289389648704	-2,82E-03	0.000432216798989568	0.00475438478888525
ME9	0.250939893902539	-2,08E-03	0.00126715686007854	0.00628906501029388
ME5	0.250258240452466	-1,99E-03	0.00130602212505586	0.00628906501029388
ME2	0.248213351303159	5,00E-03	0.00142933295688497	0.00628906501029388
ME18	0.225281574744215	-1,33E-03	0.00377065942861629	0.0121009172186179
ME16	-0.221855805560483	9,54E-04	0.00433006207781922	0.0121009172186179
ME17	0.221454355193759	-1,91E-03	0.00440033353404288	0.0121009172186179
ME10	0.202708547654939	1,68E-03	0.00909054891563437	0.0222213417937729
ME8	0.184869342357601	-1,79E-04	0.0172908965261605	0.0380399723575531
ME21	0.170814895785521	-6,65E-04	0.0277763212669345	0.0494194756824935
ME14	0.170291465707066	-1,95E-03	0.0282554147048215	0.0494194756824935
ME6	0.169278999905334	-1,36E-04	0.0292024174487462	0.0494194756824935
ME15	-0.155443145489538	-4,92E-04	0.0451480718467116	0.0709469700448326
ME12	0.128927365345801	4,19E-04	0.0963875946870615	0.135160072822786
ME11	0.126461119272407	-4,64E-03	0.102909623413526	0.135160072822786
ME4	0.123828122349462	-1,30E-04	0.110256266211857	0.135160072822786
ME19	-0.123713422968749	2,02E-03	0.110585514127734	0.135160072822786
ME0	-0.0979414925758718	-3,44E-03	0.206302121049533	0.238876140162618
ME20	0.0955122001239801	-1,21E-03	0.217761500702471	0.239537650772718
ME1	-0.0406669658911704	-1,18E-02	0.599507049132984	0.628055003853602
ME7	0.0326788248785573	-4,34E-03	0.673036335506532	0.673036335506532

PM

ME16	0.2613435049276561	-1,78E-01	0.009341739958394076	0.2730933339988925
ME29	0.22810120397082367	-1,18E-01	0.023195536318564695	0.2730933339988925
ME14	0.22546661309240473	-3,23E-01	0.02482666672717205	0.2730933339988925
ME7	0.19228916999807105	3,58E-01	0.05549554851432976	0.2787771696930863
ME15	-0.19218211126983772	3,64E-03	0.05563124387722106	0.2787771696930863
ME12	-0.1901201095087372	-2,22E-01	0.05829934302538861	0.2787771696930863
ME5	-0.18949079942944547	3,50E-03	0.0591345511470183	0.2787771696930863

ME21	-0.17676409033871732	-4,12E-03	0.07827478652579735	0.31306060777380346
ME18	-0.17268831800500406	-2,43E-03	0.08538016575649185	0.31306060777380346
ME23	0.16371433850222497	1,73E-03	0.10287004287265972	0.3201359658434896
ME4	-0.1619090712326867	-4,42E-02	0.10671198861449653	0.3201359658434896
ME31	-0.15435122127370673	-2,20E-02	0.12404812240103562	0.3295121540116228
ME2	0.15202421958851714	-3,16E-02	0.12980781824700294	0.3295121540116228
ME17	0.13805172207260125	-1,27E-01	0.16886411153606976	0.39803683433502157
ME1	0.13296272629029635	-1,73E-03	0.1850979249741938	0.40721543494322643
ME22	0.1247486905000082	-1,04E-01	0.21369737745752623	0.4300415597529466
ME25	0.12105966714345462	-1,72E-02	0.22753335949180178	0.4300415597529466
ME19	0.11924535617456304	-1,30E-01	0.23456812350160725	0.4300415597529466
ME13	0.11425575735593958	4,94E-04	0.25470678462294355	0.44238546802932305
ME10	-0.10681038060729404	-1,97E-02	0.2869468783851486	0.47346234933549514
ME30	0.08198404881378482	1,51E-02	0.41364382901028196	0.6120788924286026
ME28	-0.08114562280782994	-1,34E-01	0.41843322059133636	0.6120788924286026
ME24	0.07955015148184628	3,63E-02	0.42763699825309465	0.6120788924286026
ME3	0.07156920396391406	8,67E-04	0.4754157940028627	0.6120788924286026
ME32	-0.07107283649398415	2,41E-02	0.47848131608760436	0.6120788924286026
ME9	0.07038713800074883	-6,94E-04	0.4827338804145571	0.6120788924286026
ME20	-0.06548255231569823	-3,54E-02	0.513742650157659	0.6120788924286026
ME27	0.06461410296224095	1,40E-01	0.5193396663030567	0.6120788924286026
ME26	-0.059289227046619625	-7,72E-03	0.5543343826282524	0.6307942974735287
ME8	-0.035053765309243555	-1,04E-02	0.7266265003671234	0.7763024210692125
ME11	0.03470298748892597	-1,49E-02	0.7292537894892603	0.7763024210692125
ME6	0.028652651314889612	-4,72E-02	0.7750477908383936	0.7992680343020934
ME0	-0.022788944955584745	-5,12E-02	0.820192515668878	0.820192515668878
